# The Loss of Function of the *NODULE INCEPTION-Like PROTEIN 7* Enhances Salt Stress Tolerance in *Arabidopsis* Seedlings

**DOI:** 10.3389/fpls.2021.743832

**Published:** 2022-01-24

**Authors:** Quang Tri Le, Won Je Lee, Jun Ho Choi, Dinh Thanh Nguyen, Hai An Truong, Sang-A Lee, Suk-Whan Hong, Hojoung Lee

**Affiliations:** ^1^Department of Plant Biotechnology, College of Life Sciences and Biotechnology, Korea University, Seoul, South Korea; ^2^Department of Forest Bio Resources, National Institute of Forest Science, Suwon, South Korea; ^3^Department of Molecular Biotechnology, College of Agriculture and Life Sciences, Bioenergy Research Center, Chonnam National University, Gwangju, South Korea; ^4^Institute of Life Science and Natural Resources, Korea University, Seoul, South Korea

**Keywords:** *Arabidopsis*, *NLP7*, nitrate reductase, nitric oxide, salt stress tolerance

## Abstract

Plants acquire nitrogen, an essential macronutrient, from the soil as nitrate. Since nitrogen availability is a major determinant of crop productivity, the soil is amended with nitrogenous fertilizers. Extensive use of irrigation can lead to the accumulation of salt in the soil, which compromises crop productivity. Our characterization of *NODULE INCEPTION (NIN)-like PROTEIN 7* (*NLP7*), a transcription factor regulating the primary response to nitrate, revealed an intersection of salt stress and nitrate metabolism. The growth of loss-of-function mutant *nlp7* was tolerant to high salinity that normally reduces the fresh weight and chlorophyll and protein content of wild type (Col-0). On a medium with high salinity, the *nlp7* experienced less stress, accumulating less proline, producing less nitric oxide (NO) and reactive oxygen species (ROS), and expressing lower transcript levels of marker genes, such as *RD29A* and *COR47*, than Col-0. Nevertheless, more sodium ions were translocated to and accumulated in the shoots of *nlp7* than that of Col-0. Since *nlp7* also expressed less nitrate reductase (NR) activity, nitrate accumulated to abnormally high levels with or without salinity. We attributed the enhanced salt tolerance of *nlp7* to the balanced accumulation of nitrate anions and sodium cations. Our results suggest that nitrate metabolism and signaling might be targeted to improve salt tolerance.

## Introduction

Nitrogen is an essential macronutrient, and its availability may be a limiting factor for crop productivity. To meet the increasing demand for food, nitrogen-based fertilizers are increasingly utilized in agriculture worldwide (Nosengo, 2003), and consumption of fertilizer is expected to increase from 1.25 to 236 million tons by 2050. However, from the fertilizer applied to soil, only 30–50% of added nitrogen is actually absorbed by plants (Good and Beatty, 2011), and residual nitrogen is released into the surrounding environment, causing severe pollution and ecological imbalance through natural processes (Garnett et al., 2009). Thus, protecting the environment while increasing crop productivity is not an easy task. In addition, these competing demands must also take into account, global climate change.

The *Arabidopsis NODULE INCEPTION (NIN)-LIKE PROTEIN* (*NLP*) gene family encodes the core transcription factors that regulate nitrate signaling in plants (Wang et al., 2018). Among these, *NLP7* acts as a master regulator, controlling the primary response in nitrate signaling (Konishi and Yanagisawa, 2013; Marchive et al., 2013; Liu et al., 2017; Zhang et al., 2021). The loss-of-function *nlp7* exhibits a nitrogen-deficient phenotype as well as tolerance to drought (Castaings et al., 2009). While the vital role of *NLP7* in the transcriptional response to nitrate has been characterized, the mechanism responsible for tolerance of *nlp7* to abiotic stress has not been established.

Nitrogen metabolism also interacts with responses to salt stress. In particular, nitric oxide (NO) plays a pivotal role in responses to various abiotic stresses, such as drought and high osmotic potential. The NO accumulates with osmotic stress and is likely a product of nitrate reductase (NR) (Kolbert et al., 2010). Treatment of wheat with NO promotes stomatal closure and enhances drought tolerance (García-Mata and Lamattina, 2001), and supplying osmotically stressed wheat seedlings with NO that reduces water loss and promotes abscisic acid (ABA) accumulation (Xing et al., 2004). At low concentrations, NO positively contributes to stress signaling; however, at higher concentrations of NO, this free radical causes damage to cells (Del Rio et al., 2004).

Due to irrigation practices and climate change, soil salinization affects 1–10 billion hectares of arable land in over 100 countries (Zhang et al., 2017). In this new century, salinization is predicted to impact half of the arable land adversely. The major abiotic stressors, drought, and salinity, are already responsible for substantial crop production losses worldwide (Mahajan and Tuteja, 2005; Munns, 2011). To alleviate the effects of salt stress on crops and maximize crop productivity, an understanding of the various physiological phenomena contributing to salt tolerance at a molecular level is needed.

High salinity increases the osmotic potential of water in the soil, hindering the ability of plants to acquire water, and the water deficit ultimately leads to reduced growth (Machado and Serralheiro, 2017). Salinity also impacts plant growth by disrupting cell ion homeostasis through the toxic effects of excess sodium and chloride ions in the plant body (Munns et al., 2012) and inhibition of the uptake of essential nutrients (Paranychianakis and Chartzoulakis, 2005; Naeem et al., 2010).

Early signaling in response to salinity includes redistribution of intracellular calcium, generation of reactive oxygen species (ROS), phosphorylation by protein kinases, and accumulation of and transcriptional response to the hormone, ABA. The induction of ABA-independent genes in response to salt stress is also reported (Hussain et al., 2021). Salinity-induced signaling ultimately leads to altered physiology, such as changes in growth and development, redistribution of intracellular ions, and synthesis of compatible solutes (Acosta-Motos et al., 2017; Zhao et al., 2020).

In addition, salt stress is known to alter uptake, assimilation, and transport of nitrate in plant cells (Lin et al., 2008; Yao et al., 2008) through the inhibition of the activities of enzymes for assimilation of nitrate, including nitrate transporters and nitrate reductase (Goel and Singh, 2015). Moreover, in plants coping with the detrimental effects of high salinity, the downregulation and upregulation of *NRT1.5* and *NRT1.8*, respectively, are associated with improved tolerance to salt (Fan et al., 2007; Han et al., 2016).

From an effort to understand the response of *NLP7* to abiotic stress, we find that *nlp7* performs better than wild type (Col-0) on saline medium, although *NLP7* is induced by salinity. Moreover, the tolerance of *nlp7* to salt stress is associated with attenuated responses indicative of salt stress. We attribute the better performance of *nlp7* to the abnormal accumulation of nitrate resulting from reduced expression of NR activity in *nlp7*.

## Materials and Methods

### Plant Growth Materials

Two T-DNA-tagged mutants, namely, *nlp7-1* [SALK_026134.54.75. (SALK_026134c)] and *nlp7-2* [SALK_114886.35.50.x (CS868891)], were obtained from the Arabidopsis Biological Resource Center (ABRC), Ohio State University. The T-DNA right-border primer LBb1.3 (5′-ATTTTGCCGATTTCGGAAC-3′), the *NLP7* full-length forward primer (5′-ATGTGCGAGCCCGATGATAATTC-3′), and the *NLP7* full-length reverse primer (5′-TCACAATTCTCCAGTGCTCTCGCAG-3′) were used for PCR screening. *Arabidopsis thaliana* Col-0 was used as the wildtype. The seeds were sterilized, stored at 4°C for 3 days, and then inoculated on nitrogen-free half-strength Murashige and Skoog (MS) medium containing 2% sucrose, 0.5% phytagel, and 0.5 mM (low nitrate) or 5 mM (normal nitrate) KNO_3_ as the sole nitrogen source (pH 5.8). The seedlings were grown in a growth chamber at 23 ± 1°C under a 16/8 h light/dark cycle, 50–55 μmol photons m^–2^⋅s^–1^ photosynthetic photon flux density, and 70% humidity. After 4 days of germination, the seedlings were transferred on the same medium supplemented with 150 or 200 mM NaCl or KCl. The K^+^ concentration was adjusted to 10 mM by adding K_2_SO_4_ to all NaCl media with KNO_3_ concentration below 10 mM.

### Quantitative Reverse Transcription Polymerase Chain Reaction

The RNA was extracted from whole 9-day-old plants, as described by Lee et al. (2021). Moloney Murine Leukemia Virus (M-MLV) reverse transcriptase was used to synthesize the first-strand of complementary DNA (cDNA). Real-time reverse transcription polymerase chain reaction (RT-PCR) was conducted by using EvaGreen 2 × qPCR MasterMix (Applied Biological Materials Inc., Richmond, Canada). The housekeeping gene, *AtActin2* was used as the internal control. The primers used for PCR are listed in Supplementary Table 1.

### Chlorophyll Assay

Spectrophotometry was used to detect chlorophyll content. Briefly, chlorophyll was extracted from the investigated seedlings in the phenotypic experiments. First, the samples (100 mg) were ground to a fine powder in liquid nitrogen. The powdered samples were transferred to a 1.5 mL tube and incubated at 21°C with 700 μL of 80% acetone solution. The solutions were gently mixed in the dark for 30 min to protect chlorophyll from light damage. The mixture was then centrifuged at 3,000 rpm at 4°C for 15 min. Absorbance was measured at 663 and 645 nm. The following equations were used to estimate chlorophyll content:


Chlorophylla(mg⋅g)-1=[12.7×(A)663-2.69×(A)645]×V/1000×W

Chlorophyllb(mg⋅g)-1=[22.9×(A)645-4.86×7(A)663]×V/1000×W

Totalchlorophyll(mg⋅g)-1=[8.02×(A)663+20.20×(A)645]×V/1000×W

where, V = volume of the extract and W = fresh weight of leaves.

### Bradford Assay

After nine days of growth in the control medium, Col-0 and *nlp7* seedlings were exposed to 0, 150, or 200 mM NaCl for 1 day. Then, 500 μL of PRO-PREPTM protein extraction solution (iNtRON Biotechnology Inc., Gyeonggi, Republic of Korea) was added to the ground samples, and the mixtures were vortexed. Cell lysis was achieved by incubation at –20°C for 20–30 min, followed by centrifugation at 13,000 rpm and 4°C for 5 min. The supernatant was transferred to a fresh tube, and the samples were incubated at –20°C until used for further experiments. The protein content was determined using the BCA Protein assay kit (Merck Millipore, MA, United States). For the assay, 1 mL of bovine serum albumin solution was prepared in distilled water (10 mg⋅mL^–1^) and then diluted to 1 mg⋅mL^–1^ for use. The reaction mixtures were prepared in tubes. Next, 200 μL of the reaction mixture was transferred to a 96-well microplate, and absorbance was measured at 595 nm. Microsoft Excel was used for constructing a standard curve to calculate the protein content.

### Intracellular Nitric Oxide Detection Assay

Endogenous NO content was semi-quantitatively analyzed using a NO-specific fluorescent probe (diaminofluorescein-based dye, DAF-FM DA), as Guo et al. (2003) described with some modifications. After passing through the cell membrane, DAF-FM DA accumulates within the cell. The binding of nonfluorescent DAF-FM to intracellular NO leads to the generation of a fluorescent triazole product (Li et al., 2016). In this assay, 9-day-old seedlings were exposed to 150 mM NaCl for 6 h and then stained with 5 μM DAF-FM DA in 20 mM HEPES-NaOH (pH 7.5) for 30 min in the dark. Next, the stained seedlings were washed with the same buffer three times for 5 min each and incubated in the dark for 1 h before visualizing under a laser confocal scanning microscope (LSM 700; Zeiss, Jena, Germany). The excitation wavelength was 488 nm, and the emission wavelength was 515–565 nm.

### Nitrate Reductase Activity Assay

After 9 days of growth in the control medium, Col-0 and *nlp7* seedlings were exposed to 0, 150, or 200 mM NaCl for 1 day. The NR was isolated, and its content was determined using an NR assay kit (BC0080, SolarBio, Beijing, China). Briefly, the samples (100 mg) were extracted in 1 ml of extraction solution and centrifuged at 4,000 × *g* for 10 min. The supernatant was collected and used for further analyses. Absorbance at 520 nm was used to calculate NR activity.

### Nitrate Content Assay

Samples (50 mg) of 9-day-old Col-0 and *nlp7* seedlings grown in the control medium and then treated with 0, 150, or 200 mM NaCl for 1 day were collected for nitrate content measurements as described by Zhao and Wang (2017). The pretreated samples were ground in liquid nitrogen. Then, 1 mL of distilled water was added to the samples, followed by boiling the samples for 20 min. The mixture was centrifuged at 13,000 rpm at 4°C for 10 min. Next, 100 μl of the supernatant was mixed with 400 μl of sulfosalicylic acid in a 15 ml Falcon tube and incubated at room temperature for 30 min. Then, 9.5 ml of 8% NaOH solution was added to the samples, and the mixture was cooled at 4°C for 5 min. Nitrate content was calculated based on absorbance at 410 nm.

### Chloride Content Assay

Chloride content was analyzed using the Ferricyanide method described by Hunt (1982) and Pruefer (2001). Briefly, after 9 days of growth in the control medium, Col-0 and *nlp7* seedlings were exposed to 0, 150, or 200 mM NaCl for 1 day. Samples (50 mg) of pretreated seedlings were collected and ground in liquid nitrogen. Collected samples were incubated in 5 ml of 0.5 M HNO_3_ solution in an oven at 80*^o^*C for 1 h. Then, solid materials were allowed to settle to the bottom of the vial. A stock solution containing mercuric thiocyanate solution, Hg(SCN)_2_ (4.17 g/l distilled water), and ferric nitrate solution, Fe(NO_3_)_3_•9H_2_O (202 g/l DW), was prepared. Then, a combined color regent was made by adding 150 ml stock Hg(SCN)_2_ solution to 150 ml stock Fe(NO_3_)_3_ solution and diluted to 1 L with distilled water. Next, 1 ml of the supernatant was mixed with 3 ml of the color regent in a 15 ml Falcon tube. Chloride content was calculated from a standard curve obtained with Cl^–^ standard solutions using a spectrophotometer with the wavelength set at 480 nm.

### ^15^N-Uptake Assay

Nitrate uptake was analyzed using ^15^NO_3_^–^, as previously described by Lin et al. (2008). Briefly, seedlings were grown in a nitrogen-free half-strength MS medium with or without 200 mM NaCl for 9 days, treated with 0.1 mM CaSO_4_ for 1 min, and then transferred into a fresh nutrient solution supplemented with 20 mM K^15^NO_3_^–^ (99% atom), as the sole N source, for 1 h. The seedlings were treated with 0.1 mM CaSO_4_ for 1 min once again. The roots were dried at 70°C to a constant weight and ground. The ^15^N content was analyzed using a continuous-flow isotope mass spectrometer (Thermo-MAT253) coupled to an elemental analyzer (Flash 2000 HT, Thermo Fisher Scientific Inc., MA, United States).

### Promoter Activity Assay

An approximately 1.45 kb genomic fragment of the *NLP7* promoter (–231 to –1,682) was PCR-amplified using the *NLP7-proGUS* forward (5′-GGGCCAACTATAGAGGAATGGT-3′) and reverse (5′-ACAATACAACTGTGCCCCAAAT3′) primers. After sequencing, the promoter fragment was cloned in front of the reporter *GUS* gene in the binary vector, pMDC162. This vector was then introduced into *Agrobacterium tumefaciens* and finally transformed into Col-0 using the floral dip method (Clough and Bent, 1998). Putative transformants were selected on half-strength MS media supplemented with hygromycin B (25 mg⋅L^–1^). The β-glucuronidase (GUS) staining was performed as described by Jefferson et al. (1987), with some modifications. Briefly, 9-day-old *NLP7*p*::GUS* transgenic seedlings were pretreated under the abovementioned conditions for 1 day, followed by GUS staining. The Leica EZ4D microscope (Leica Microsystems^1^) was used to examine the stained seedlings.

### Proline Content Measurement

Proline content was measured as described previously (Bates et al., 1973). Briefly, proline was isolated from 100 mg of plant leaves by grinding in 1 ml of 3% sulfosalicylic acid. A 200 μl aliquot of the extract was allowed to react with 100 μl ninhydrin (80% glacial acetic acid, 6.8% phosphoric acid, and 70.17 mM ninhydrin) for 60 min at 100°C. An ice bath was used to stop the reaction. Then, the reaction mixture was treated with 200 μl of toluene and vortexed. The absorbance of the toluene layer was measured at 520 nm using a UV/VIS spectrophotometer. Finally, proline content was estimated by extrapolation on a standard curve and calculated on the FW basis as follows: [(ng proline ml^–1^× ml extraction buffer)/115.5 ng nmol] g^–1^ sample = nmol proline g^–1^ FW material.

### Malondialdehyde Content Measurement

After 24 h of salt treatment, samples were harvested, and the malondialdehyde (MDA) content was assayed according to the study by Zhang et al. (2020) with small modifications. Seedlings were ground in liquid nitrogen, homogenized in 1.5 ml of 20% (w/v) trichloroacetic acid (TCA), and centrifuged at 10,000 × *g* for 5 min. To a 1 ml aliquot of the supernatant, 2 mL of thiobarbituric acid solution [0.5% (w/v) in 20% TCA] was added. Then, the mixture was heated at 95°C for 15 min, rapidly cooled in an ice bath, and centrifuged at 12,000 × *g* for 10 min. The absorbance of the supernatant was measured at 450, 532, and 600 nm, and the MDA content was calculated using the following equation:


Concentration(mmolL)-1=6.453×(OD-532OD)600-0.563×OD450

where, OD = optical density.

### Measurement of Abscisic Acid Content

Using 200 mg samples of seedlings treated with 0, 150, or 200 mM NaCl for 1 day, the ABA content was determined as described previously (Liu et al., 2014; Jeong et al., 2018) using a commercial kit (Phytodetek Elisa kit, PDK 09347, Agdia, Inc., Elkhart, Indiana, United States) according to the manufacturer’s instructions.

### Na^+^ and K^+^ Ion Content Measurement

The cation content was determined as described by Choi and Gilroy (2015). Briefly, 100 mg samples of Col-0 and *nlp7* seedlings germinated and grown on nitrogen-free half-strength MS medium containing 5 mM KNO_3_ and treated with 200 mM NaCl for 6–24 h were used. The pretreated seedlings were washed multiple times with deionized water to remove any Na^+^ and K^+^ on the surface. In 20 mL glass test tubes, the samples were digested with 0.6 mL of concentrated HNO_3_ at 150°C until the plant tissues were entirely dissolved. Next, 0.4 mL of HClO_4_ was added, and the samples were continuously digested further at 180°C until the total sample volume dropped below 0.5 mL. Then, the extracts were cooled down to room temperature, and the final volume was adjusted to 5 mL. The Na^+^ and K^+^ content were determined using ICP-OES (Agilent Technologies Inc., CA, United States) from a standard curve obtained with Na^+^ and K^+^ standard solutions. Moreover, the Na^+^-to-K^+^ ratio was calculated based on the measured Na^+^ and K^+^ content.

### Gene Accession Numbers

The ABRC accession numbers of gene sequences used in the present article are provided in parentheses: *NLP7* (AT4G24020), *NLP6* (AT1G64530), *NRT1.1* (AT1G12110), *HY5* (AT5G11260), *COR47* (AT1G20440), *RD29A* (AT5G52310), *NIA1* (AT1G77760), *NIA2* (AT1G37130), *NRT1.5* (AT1G32450), *NRT1.8* (AT4G21680), *NCED3* (AT3G14440), *BG1* (AT3G57270), and *BG2* (AT3G57260), *ACTIN2* (AT3G18780).

### Statistical Analyses

To obtain reliable results, each experiment was independently repeated at least three times. All statistical analyses were performed using one-way ANOVA, followed by Tukey’s test, for the comparison of means with a 95% confidence level. Different letters (a, b, c, …) indicate significant differences at *P* < 0.05. Error bars represent standard deviation (SD).

## Results

### Salt-Induced *NLP7* Conditions Tolerance to Salinity

**FIGURE 1:**
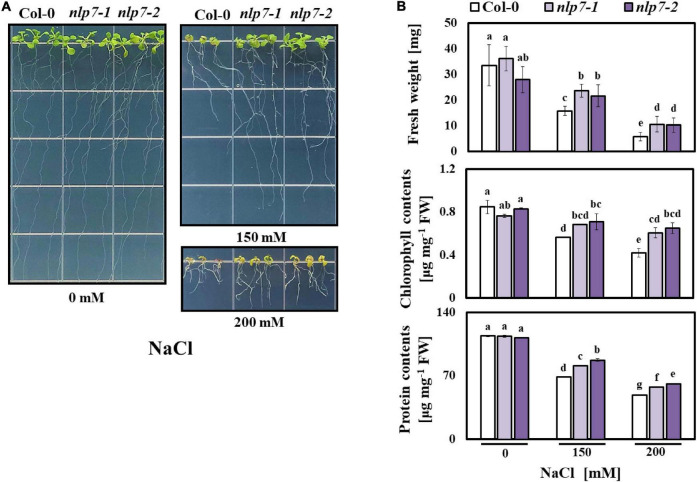
The *nlp7* mutants showed enhanced salt tolerance. **(A)** Comparison of the growth performance of Col-0 and *nlp7* plants under salt stress. Four-day-old Col-0, *nlp7-1*, and *nlp7-2* seedlings were germinated and grown on a nitrogen-free half-strength MS medium containing 5 mM KNO_3_ and then transferred to a media supplemented with 0, 150, or 200 mM NaCl. After 2 weeks, each phenotype was confirmed. **(B)** Quantification of fresh weight, chlorophyll content, and protein content of Col-0, *nlp7-1*, and *nlp7-2* seedlings in response to salt stress. Error bars represent the standard deviation of three biological replicates. Different letters (a, b, c, or d) within a treatment group indicate significant differences in the two-way ANOVA (*P* ≤ 0.05, Tukey’s test).

A previous study showed that *nlp7* exhibits tolerance to drought (Castaings et al., 2009), which led us to examine the tolerance of this mutant to salinity. Thus, we assessed the performance of *nlp7* grown in stressful concentrations of NaCl. Four-day-old seedlings of Col-0, *nlp7-1*, and *nlp7-2* were transferred from agar medium without added NaCl to medium supplemented with 0, 150 and 200 mM NaCl. After 2 weeks, there was no difference in the growth performance of Col-0 and *nlp7* in the absence of NaCl; however, on high salinity, *nlp7* showed a higher tolerance. Although all plants grew less on saline medium, *nlp7* grew noticeably better than Col-0 (Figure 1A). Moreover, *nlp7* exhibited higher fresh weight, chlorophyll content, and protein content than Col- 0 in the presence of 150 mM NaCl, and these differences were still greater at a higher NaCl concentration (200 mM) (Figure 1B). In contrast, the performance of Col-0 and *nlp7* grown on medium supplemented with KCl (100 and 200 mM) was indistinguishable although all plants appeared more sensitive to KCl than to NaCl (Supplementary Figures 1A,B).

**FIGURE 2:**
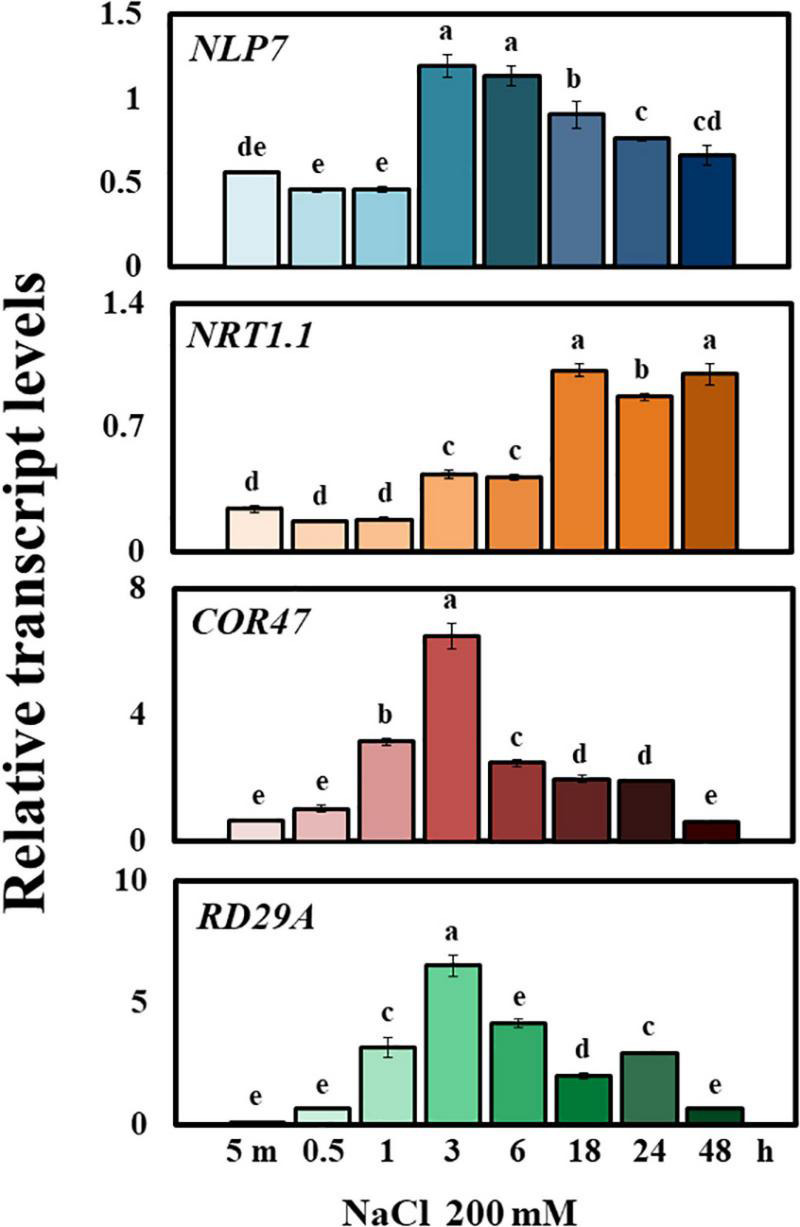
*NLP7* transcript levels were elevated in response to external salt treatment. Relative transcript levels of *NLP7*, *NRT1.1*, *COR47*, and *RD29A* in Col-0 and *nlp7* plants, as determined using qPCR. The seeds were germinated and grown on a nitrogen-free half-strength MS medium containing 5 mM KNO_3_ for 9 days and then treated with 200 mM NaCl for 5 min to 48 h. *AtActin2* was used as the internal control. Error bars represent the standard deviation of three independent replicates. Different letters (a, b, c, d, or e) within a treatment group indicate significant differences in the two-way ANOVA (*P* ≤ 0.05, Tukey’s test).

To examine whether the expression of *NLP7* responds to salinity, we measured its transcript levels in Col-0 exposed to NaCl (200 mM) at time points of up to 48 h (5 min, 0.5 h, 1 h, 3 h, 6 h, 18 h, 24 h, and 48 h, in Figure 2). The expression of *NLP7* initially remained low from 5 min to 1 h after exposure to NaCl, abruptly peaked at 3 and 6 h and then gradually declined out to 48 h. Transcription factors *NLP7* and its close homolog *NLP6* serve vital functions in nitrate signaling and, in particular, promote expression of *NITRATE TRANSPORTER 1.1* (*NRT1.1/NPLC 3.8*, Konishi and Yanagisawa, 2013; Marchive et al., 2013). *NRT1.1* encodes a transceptor (dual affinity transporter and sensor) involved in nitrate uptake and nitrate-dependent gene regulation (Huang et al., 1996; Wang et al., 1998; Liu et al., 1999; Ho et al., 2009). While the transcriptional response of *NLP7* to NaCl peaked by 3 h, the transcript level of *NRT1.1* was maximally induced only after 18 h. The induced expression of stress marker genes *COR47* and *RD29A* confirmed that salt stress was successfully caused in the present study (Figure 2).

### The Promoter of *NLP7* Responds to Salt and Osmotic Stress

**FIGURE 3:**
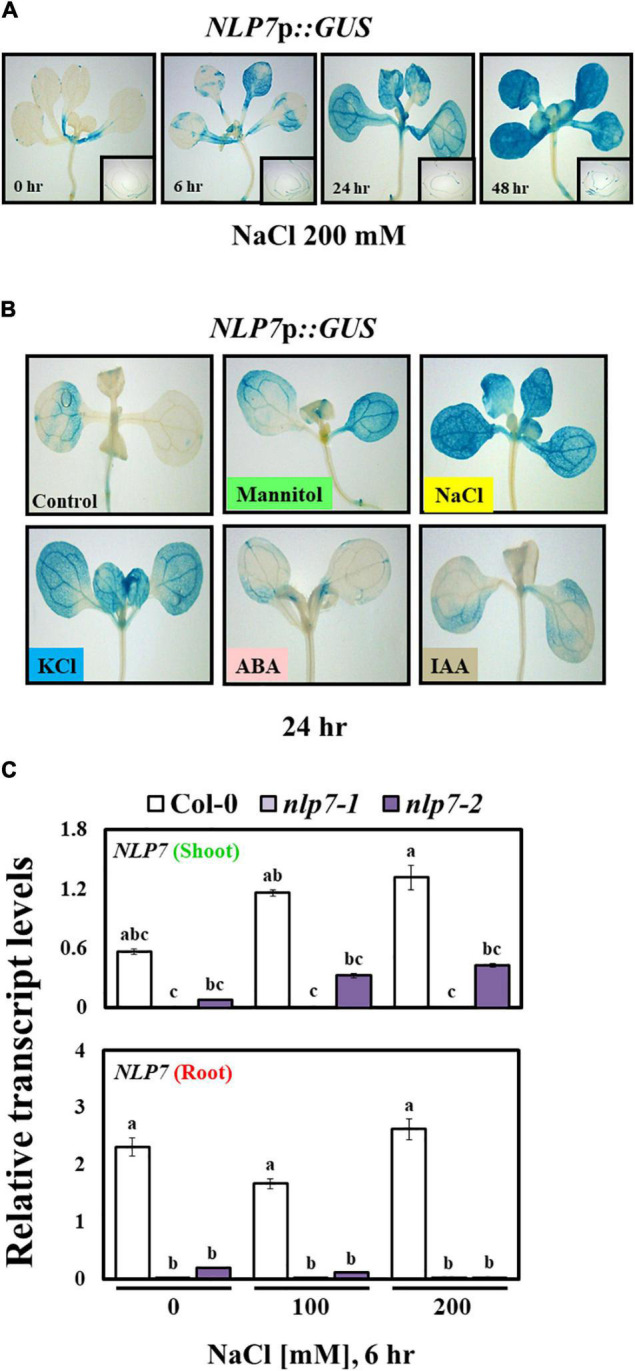
*NLP7* promoter activity was induced by salt, mannitol, and KCl. **(A)** Histochemical β-glucuronidase (GUS) staining of transgenic *Arabidopsis* seedlings harboring the *NLP7*p*::GUS* fusion construct. The seedings were germinated and grown on half-strength MS medium containing 5 mM KNO_3_ for 9 days and then treated with 200 mM NaCl for 0, 6, 24, or 48 h. **(B)** Histochemical GUS staining of the 9-day-old seedlings grown on a nitrogen-free half-strength MS medium containing 5 mM KNO_3_ in response to 300 mM mannitol, 200 mM NaCl, 200 mM KCl, 5 μM ABA, and 0.5 μM IAA for 24 h; untreated seedlings were used as controls. **(C)** Relative transcript levels of *NLP7* in the shoots and roots of 9-day-old Col-0, *nlp7-1*, and *nlp7-2* seedlings following exposure to 0, 150, or 200 mM NaCl for 6 h. *AtActin2* was used as the internal control. Error bars represent the standard deviation of three independent replicates. Different letters (a, b, or c) within a treatment group indicate significant differences in the two-way ANOVA (*P* ≤ 0.05, Tukey’s test).

Since salinity enhanced the transcript levels of *NLP7*, we examined the transcriptional regulation of the promoter of *NLP7* using plants stably transformed with *NLP7*p::*GUS*, a fusion of genomic sequence (1.45 kb) upstream of *NLP7* to the reporter gene, *GUS*. Following exposure to 200 mM NaCl, the GUS activity of transformants gradually increased with time under stress (at 6, 24, and 48 h); and the activity was higher in shoots than in the roots (Figure 3A). To determine whether promoter activity specifically responded to salt stress, we examined other treatments. Similar to NaCl, both mannitol (300 mM) and KCl (200 mM) activated *NLP7*p::*GUS*, whereas ABA (5 μM) and IAA (0.5 μM) did not activate *NLP7*p::*GUS* (Figure 3B). In a previous study, GUS activity of a reporter gene similar to *NLP7*p::*GUS* increased in the guard cells of transgenic plants, which induces drought resistance (Castaings et al., 2009). High salinity affects plants through two mechanisms, osmotic stress and ion toxicity (Ma et al., 2020). The early response of plants to salt stress is similar to drought stress due to a decline in the water potential (Navarro et al., 2008; Ma et al., 2020). Our results show that the promoter of *NLP7* responded to osmotic stress and salinity.

We further examined the transcript accumulation of *NLP7* separately in the shoots and the roots in response to salt stress. Following treatment with 150 and 200 mM NaCl, transcripts of *NLP7* substantially accumulated in shoots and not in the roots of Col-0 in response to NaCl, which was consistent with the pattern of GUS activity in transgenic plants (Figure 3C). Similarly, following treatment with 100 and 200 mM KCl, the transcript levels of *NLP7* appreciably increased in the shoots and not in the roots (Supplementary Figure 2). Considering that *NLP7* is a transcriptional regulator of nitrate response and a member of a family of homologous genes, we examined how *nlp7* mutants affect the expression of *NLP6*. The transcript level of *NLP6* was unaffected by salt stress in both the shoots and the roots, and the levels were no different between Col-0 and *nlp7* with or without salt stress (Supplementary Figure 3).

### The *nlp7* Accumulated More Nitrate Than Col-0 in the Shoots

**FIGURE 4:**
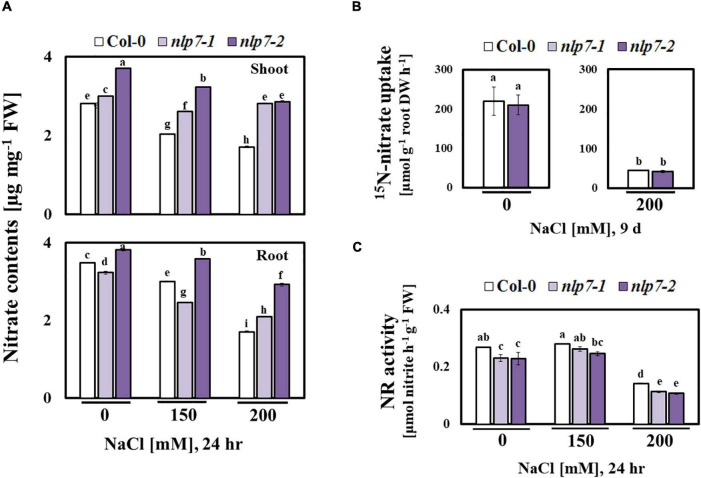
Nitrate content was elevated, and nitrate reductase activity was altered in *nlp7* mutants under salt stress. **(A)** The nitrate content of the shoots and the roots of 9-day-old Col-0, *nlp7-1*, and *nlp7-2* seedlings was measured. The seeds were germinated and grown on nitrogen-free half-strength MS medium containing 5 mM KNO_3_ for 9 days and then treated with 0, 150, and 200 mM NaCl for 24 h. Error bars represent the standard deviation of three independent replicates. **(B)** ^15^NO_3_ uptake in the roots of Col-0 and *nlp7-2* seedlings. The seedlings were germinated and grown on a nitrogen-free half-strength MS medium with or without 200 mM NaCl for 9 days and then transferred to a medium containing 20 mM of ^15^NO_3_^–^ and kept for 1 h. Error bars represent the standard deviation of three independentreplicates. **(C)** Comparison of nitrate reductase activity under salt stress between 9-day-old Col-0 and *nlp7* seedlings. Error bars represent the standard deviation of three independent replicates. Different letters (a, b, c, d, e, f, g, h, or i) within a treatment group indicate significant differences in the two-way ANOVA (*P* ≤ 0.05, Tukey’s test).

**FIGURE 5:**
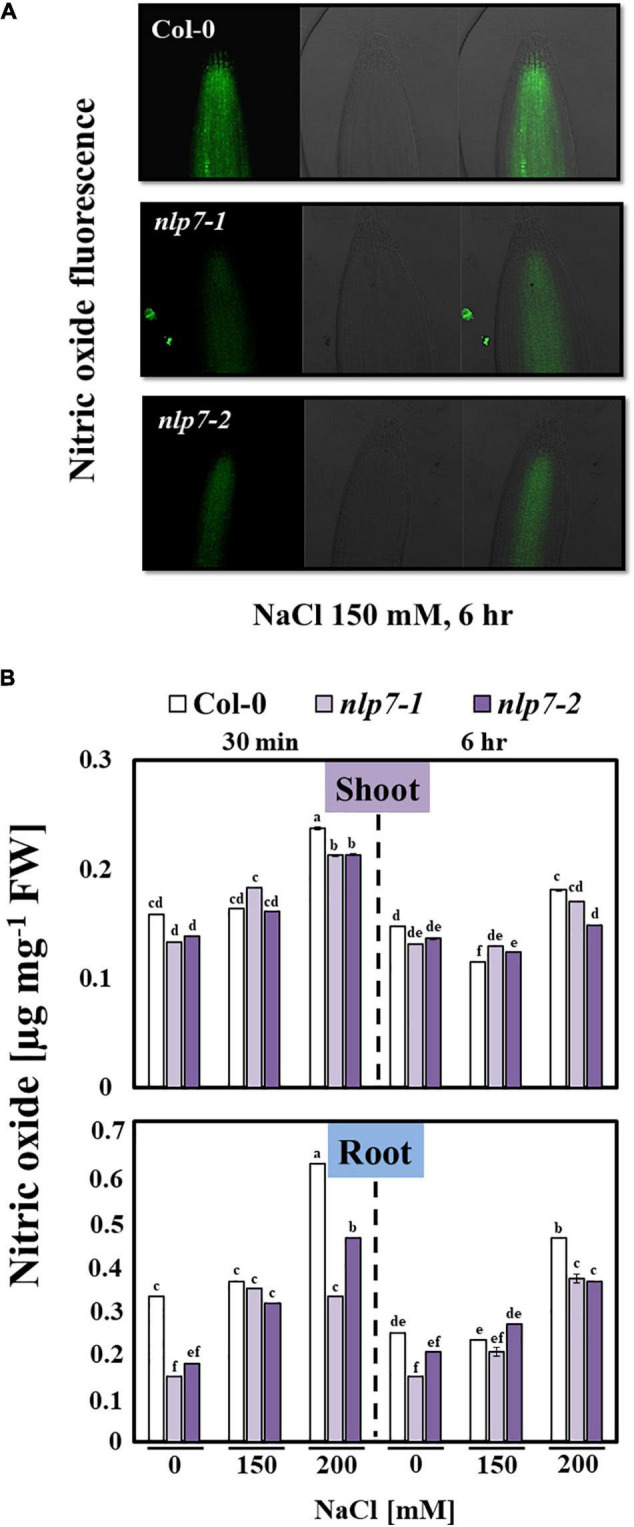
The *nlp7* mutants showed reduced nitric oxide (NO) content under salt stress. **(A)** The NO accumulation in the root tips of Col-0 and *nlp7* seedlings following exposure to 150 mM NaCl. The Col-0, *nlp7-1*, and *nlp7-*2 seedlings were germinated and grown on a nitrogen-free half-strength MS medium containing 5 mM KNO_3_ for 9 days and then treated with 200 mM NaCl for 6 h. The roots of the test seedlings were incubated with 5 μM of DAF-FM DA in 20 mM HEPES-NaOH (pH 7.5) for 30 min in the dark, and the NO-associated fluorescence was visualized using a laser confocal scanning microscope (LSM 700; Zeiss, Jena, Germany). The excitation wavelength was 488 nm, and emission wavelength was 515–565 nm. Images on the left were obtained under green fluorescence, and the ones in the middle were obtained under bright-field microscopy; the images of green fluorescence and bright field microscopy were merged and are shown on the right. **(B)** The NO content of the shoots and the roots of Col-0, *nlp7-1*, and *nlp7-*2 seedlings was measured following treatment with 0, 150, or 200 mM NaCl for 30 min to 6 h. Error bars represent the standard deviation of three independent replicates. Different letters (a, b, c, d, e, or f) within a treatment group indicate significant differences in the two-way ANOVA (*P* ≤ 0.05, using Tukey’s test).

Since NLP7 is involved in nitrate signaling (Zhao et al., 2018), we examined the possible role of NO, a signaling molecule for salt stress tolerance (Nabi et al., 2019), in the enhanced tolerance of *nlp7* plants to salt stress. NO is enzymatically generated from nitrate as a byproduct of NR (Meyer et al., 2005). Thus, reduced NO levels may result from the reduced activity of NR in *nlp7* (Figure 4). Fluorescence of the dye, DAF-FM DA was used to measure the endogenous NO levels *in planta* (Figure 5A). The roots of *nlp7* showed lower NO levels following treatment with 0, 150, or 200 mM NaCl for 30 min to 6 h than those of Col-0 (Figure 5B).

To examine the nitrate content, we separately collected the shoot and root tissues of Col-0 and *nlp7* and found no difference in the nitrate content of Col-0 and *nlp7* in the absence of salt stress; however, the nitrate content of shoots (and not roots) of *nlp7* was higher than Col-0 under high salinity (Figure 4A). Since plants could accumulate higher chloride anion under high salt stress, we also measured the chloride content and found no difference between Col-0 and *nlp7* (Supplementary Figure 4). Moreover, the nitrate uptake of *nlp7* and Col-0 was similar following treatment with ^15^N-labeled KNO_3_ with or without 200 mM NaCl (Figure 4B). Likewise, Yu et al. (2016) have reported a similar nitrate uptake by *nlp7* and Col-0 plants in the absence of salinity.

Importantly, the transcription factor, NLP7 binds to the nitrate response element, a *cis*-element, in the promoter region of *NIA1*, encoding a critical component of NR, and regulates its transcription (Konishi and Yanagisawa, 2019). Thus, we measured the NR activity of Col-0 and *nlp7* under salt stress. As shown in Figure 4C, the NR activity was lower in *nlp7* than Col-0, suggesting that the accumulation of nitrate in *nlp7* may be a consequence of reduced consumption by NR.

**FIGURE 6:**
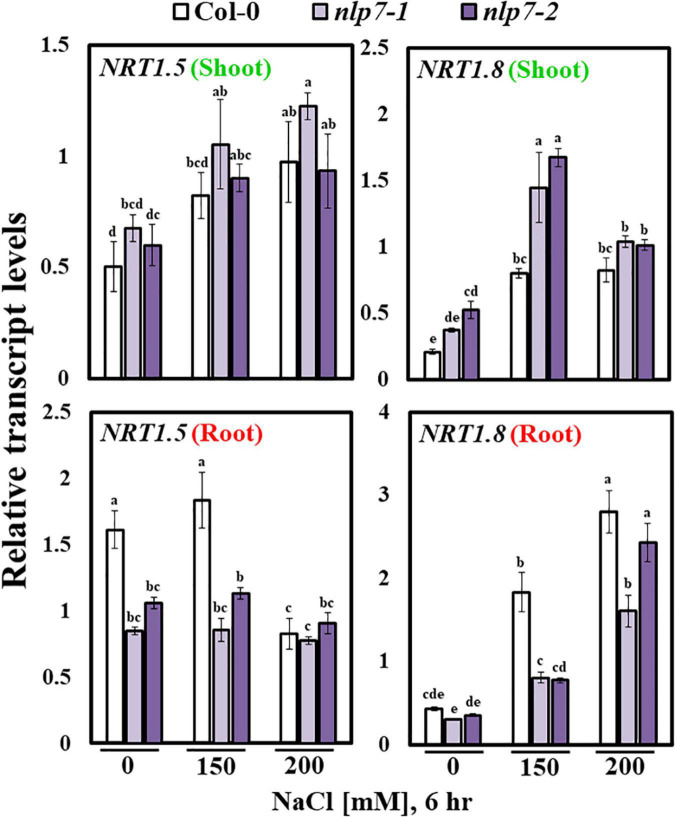
Transcript levels of nitrate transporter genes were altered in *nlp7* mutants. The relative transcript levels of the nitrate transporter genes, *NRT1.5* (transporter of nitrate from the roots to the xylem) and *NRT1.8* (responsible for nitrate unloading from the xylem and transport to the surrounding shoot organs) in the shoots and the roots of Col-0 and *nlp7* seedlings, as determined using qPCR. Nine-day-old seedlings were germinated and grown on a nitrogen-free half-strength MS medium containing 5 mM KNO_3_; treated with 0, 150, or 200 mM NaCl for 6 h; and then dissected into shoot and root parts. *AtActin2* was used as the internal control. Error bars represent the standard deviation of three independent replicates. Different letters (a, b, c, d, e, f, g, or h) within a treatment group indicate significant differences in the two-way ANOVA (*P* ≤ 0.05, Tukey’s test).

To explain how nitrate is taken up by the roots could have accumulated in the shoots of *nlp7*, we compared the transcript levels of various nitrate transporter genes in *nlp7* and Col-0. In particular, we found that the expression of *NRT1.5*, encoding a transporter that uploads nitrate into the xylem (Raddatz et al., 2020), was noticeably reduced in the roots of *nlp7* treated with 0 and 150 mM NaCl; whereas, the expression of *NRT1.8*, which unloads nitrate from the xylem (Raddatz et al., 2020), was elevated in the shoots and reduced in the roots of *nlp7* (Figure 6). These results suggest that the excess nitrate accumulation in the shoots of *nlp7* may be due to an enhanced expression of *NRT1.8*.

### Nitrate-Driven Ion Homeostasis May Contribute to the Enhanced Salt Tolerance of *nlp7* Plants

**FIGURE 7:**
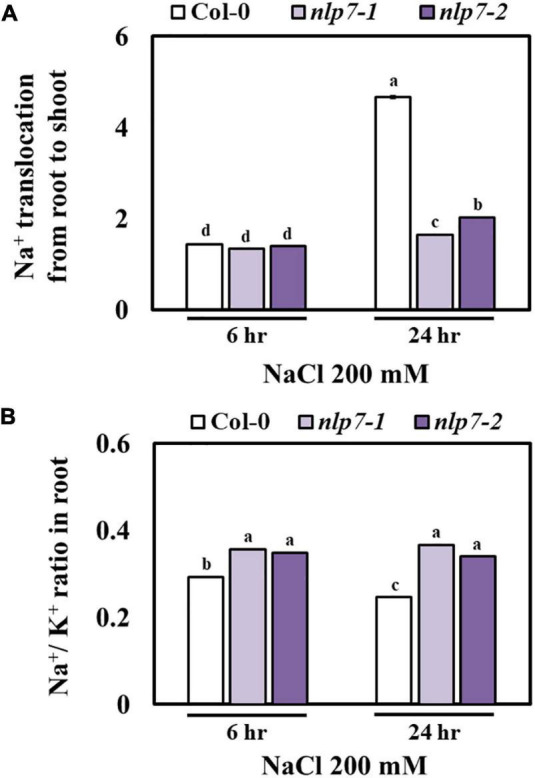
Na^+^ translocation to the shoots of *nlp7* mutants was decreased under salt stress. **(A)** The Na^+^ translocation from the roots to the shoots in Col-0, *nlp7-1*, and *nlp7-2* seedlings in response to salt stress. The seeds were germinated and grown on a nitrogen-free half-strength MS medium containing 5 mM KNO_3_ and then treated with 200 mM NaCl for 6–24 h. Na^+^ translocation from the roots to the shoots was estimated based on the Na^+^ and K^+^ content of the roots and the shoots using ICP-OES (Agilent) calculated from a standard curve obtained with Na^+^ and K^+^ standard solutions. **(B)** The Na^+^-to-K^+^ ratio was calculated based on the Na^+^ and K^+^ content of the roots and the shoots of salt-treated Col-0 and *nlp7* seedlings. Error bars represent the standard deviation of three independent replicates. Different letters (a, b, or c) within a treatment group indicate significant differences in the two-way ANOVA (*P* ≤ 0.05, Tukey’s test).

Next, we addressed whether enhanced tolerance to salt was due to the reduced accumulation of Na^+^ in *nlp7*. The rate of translocation of Na^+^ from the roots to the shoots was measured at 6 h and 24 h after exposure to 200 mM NaCl. At 6 h, the rate was no different in Col-0 and *nlp7*; however, at 24 h, the rate increased substantially in Col-0 and not *nlp7* (Figure 7A). In contrast to shoots, the accumulation of Na^+^ and K^+^ in roots under salt stress was lower in Col-0 and higher in *nlp7* (Supplementary Figure 5). A higher ratio of Na^+^/K^+^ in the roots possibly accounted for the reduced root growth (Figure 7B) of *nlp7*. In addition to being an anabolic source of nitrogen, nitrate is a persistent anion in cells and contributes to the ion homeostasis of plants by balancing the excessive Na+ cations from salt stress (Raddatz et al., 2020).

**FIGURE 8:**
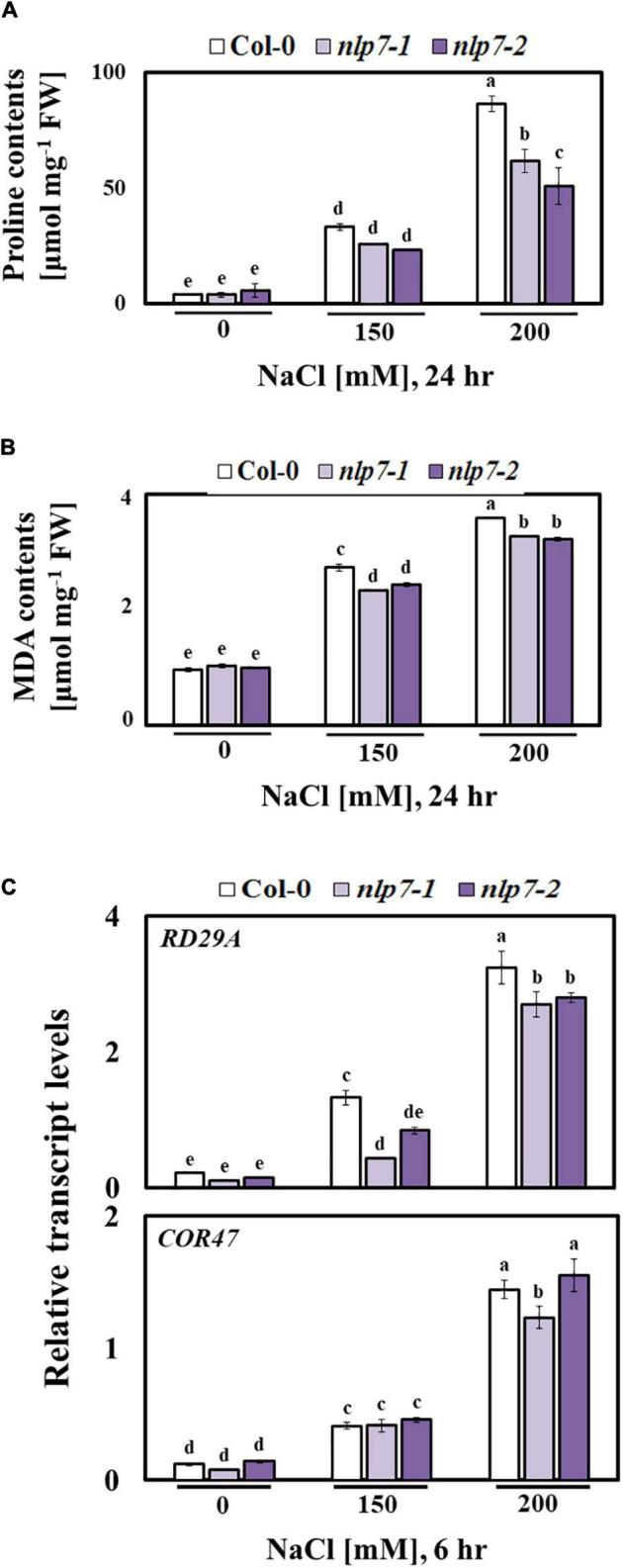
The *nlp7* mutants showed reduced proline and malondialdehyde (MDA) content and decreased relative transcript levels of stress marker genes. **(A, B)** Proline and MDA content of 9-day-old Col-0 and *nlp7* seedlings germinated and grown on a nitrogen-free half-strength MS medium containing 5 mM KNO_3_ and then treated with 0, 150, or 200 mM NaCl for 24 h. Error bars represent the standard deviation of three independent replicates. **(C)** Relative transcript levels of *RD29A* and *COR47* (stress marker genes) in 9-day-old Col-0, *nlp7-1*, and *nlp7-2* seedlings in response to salt stress, as determined using qPCR. The seeds were germinated and grown on a nitrogen-free half-strength MS medium containing 5 mM KNO_3_ for 9 days and then treated with 0, 150, or 200 mM NaCl for 6 h. *AtActin2* was used as the internal control. Error bars represent the standard deviation of three independent replicates. Different letters (a, b, c, d, or e) within a treatment group indicate significant differences in the two-way ANOVA (*P* ≤ 0.05, Tukey’s test).

Proline typically accumulates in plants stressed by salt or osmoticum (Nanjo et al., 1999; Maiale et al., 2004; Sannazzaro et al., 2007), and MDA accumulates in response to lipid peroxidation resulting from salt stress (Wu et al., 2017; Zhou et al., 2017). Interestingly, the levels of proline and MDA were lower in *nlp7* than in Col-0 following treatment with 0, 150, or 200 mM NaCl (Figures 8A,B). Moreover, the transcript levels of the stress marker genes, such as *RD29A* and *COR47*, were slightly lower in *nlp7* or similar to those in Col-0 (Figure 8C).

Yang et al. (2018) showed that *HY5* is essential for tolerance to salt stress, so we assessed the expression of *HY5*. In the shoots, the transcripts of *HY5* similarly accumulated in Col-0 and *nlp7* under salt stress, while in the roots, the level of transcripts was lower in Col-0 than in *nlp7* (Supplementary Figure 6). This finding suggests that a nitrate-dependent reduction in the accumulation of Na^+^ contributes to tolerance of *nlp7*to salt.

### Under High Salinity, Abscisic Acid Accumulated Less in Col-0 Than in *nlp7*

**FIGURE 9:**
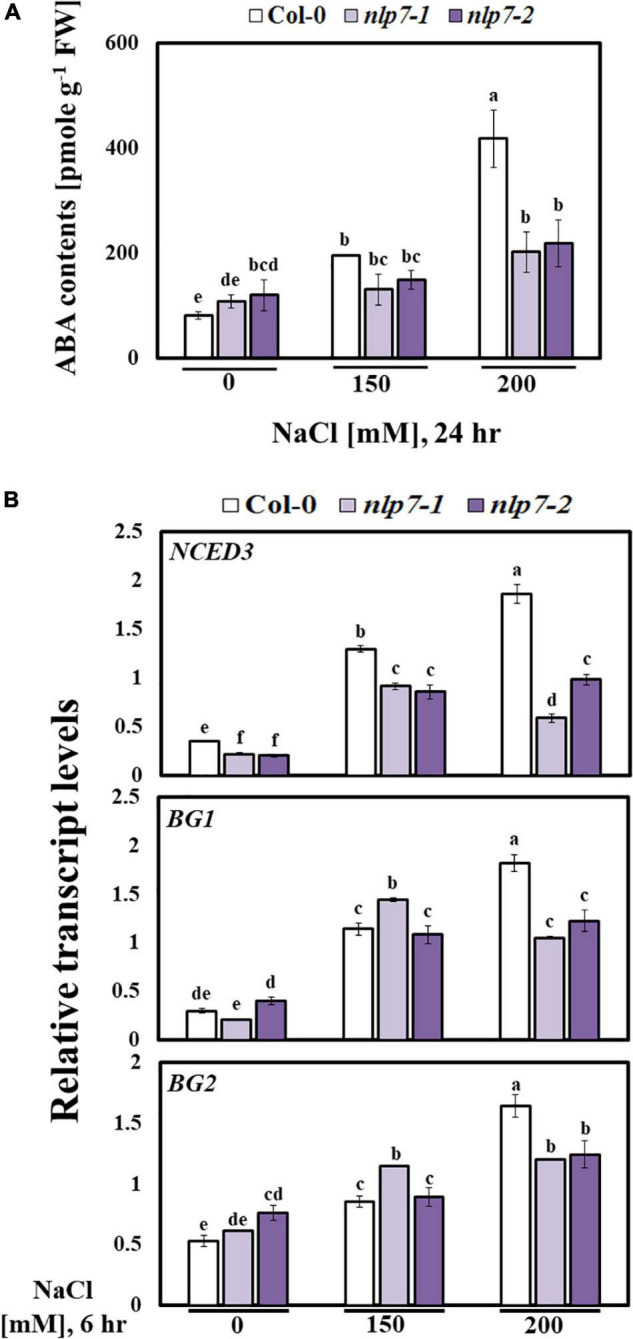
Abscisic acid accumulation was decreased in the *nlp7* mutant under salt stress. **(A)** Comparison of the abscisic acid (ABA) content between Col-0 and *nlp7* plants. Nine-day-old Col-0, *nlp7-1*, and *nlp7-2* seedlings were germinated and grown on a nitrogen-free half-strength MS medium containing 5 mM KNO_3_ and then treated with 0, 150, or 200 mM NaCl for 24 h. Error bars represent the standard deviation of three independent replicates. **(B)** Comparison of the relative transcript levels of the ABA-related genes, such as *NCED3*, *BG1*, and *BG2*, in Col-0 and *nlp7* plants. The seedlings were grown, treated, and analyzed as described in Figure 8C. *AtActin2* was used as the internal control. Error bars represent the standard deviation of three independent replicates. Different letters (a, b, c, d, e, or f) within a treatment group indicate significant differences in the two-way ANOVA (*P* ≤ 0.05, Tukey’s test).

Plant hormones play diverse roles in plants under stress. Among these, the signaling of ABA plays crucial roles in response to abiotic stress (Vishwakarma et al., 2017), and ionic and osmotic stress activates the ABA biosynthesis (Kim et al., 2010; Sah et al., 2016). To explore the involvement of *NLP7* in salinity-triggering ABA biosynthesis, we measured the ABA content of Col-0 and *nlp7* in response to added NaCl. Following treatment with 150 mM NaCl, nearly 30% less ABA accumulated in *nlp7* than in Col-0, and this difference was two-fold greater following treatment with 200 mM NaCl (Figure 9A). Moreover, we examined the expression of genes involved in ABA biosynthesis or metabolism in 9-day-old seedlings of Col-0, *nlp7-1*, and *nlp7-2* following exposure to 0, 150, or 200 mM NaCl for 6 h (Figure 9B). The transcript levels of *9-cisepoxycarotenoid dioxygenase* (*NCED3*), encoding a key enzyme in ABA biosynthesis (Urano et al., 2009; Liu et al., 2016), was markedly reduced in *nlp7*; and, the transcript level of *ß- GLUCOSIDASE 1* (*BG1*) and *ß-GLUCOSIDASE 2* (*BG2*), involved in the conversion of inactive ABA-GE (located in the endoplasmic reticulum) to free active ABA (Xu et al., 2012), was reduced in *nlp7* following treatment with 200 mM NaCl. These results suggest that *nlp7* suffered less severe damage from high salinity because less ABA accumulated in comparison to Col-0 (Figure 9A). As shown in Supplementary Figure 7, any excess of ABA would be inappropriate for attaining maximal biomass. Thus, ABA content must be optimized for maximized salt tolerance (Supplementary Figures 7A,B).

## Discussion

The *NLP7* is one of the extensively studied genes involved in nitrogen signaling. This gene belongs to the nodule inception (NIN) family (Castaings et al., 2009; Yu et al., 2016; Liu et al., 2017). NIN proteins harbor a 60-amino acid domain, called the RWPRK domain, which is similar to the DNA-binding domain of bZIP8 and bHLH/Z9 transcription factors (Schauser et al., 2005). Since NLP7 is a positive regulator of nitrogen signaling, we initially speculated that NLP7 could play a positive role under salt stress and could improve plant stress tolerance. However, the *nlp7* plants not only weighed more than Col-0 when raised under high-salinity conditions, but they also showed higher content of chlorophyll and protein per unit weight, indicating that the loss of NLP7 appears to be in fact beneficial to the salt tolerance of *Arabidopsis* seedlings (Figure 1). A similar observation was also made by another research group (Castaings et al., 2009). Therefore, we further explored why the *nlp7* plants are more resistant to salt stress than Col-0. The *NLP7* seems to be associated with salt stress signaling because its transcript levels were apparently increased in response to salt stress (Figure 2). Moreover, the *NLP7* promoter activity was increased not only by NaCl but also by KCl (Figure 3B).

Recently, NLP6 and NLP7, along with teosinte branched1/cycloidea/proliferating cell factor1-20 (TCP20), were shown to bind the promoter of the *NIA1* gene under nitrogen starvation (Guan et al., 2017). Moreover, the NIT2 protein in *Chlamydomonas reinhardtii*, which is structurally related to the plant NIN proteins, activates NR by binding its promoter (Camargo et al., 2007). As expected, under salt stress, the activity of NR was lower in *nlp7* plants than in Col-0 (Figure 4C), although nitrate uptake was equivalent between the two; thus, the reduced NR activity of *nlp7* plants does not appear to be due to the decreased nitrate uptake under salt stress (Figure 4B). Contrary to these results, nitrate content was higher in *nlp7* plants, perhaps due to the decreased NR activity (Figure 4C). Both the roots and the shoots of the *nlp7* plants accumulated more nitrate than Col-0 (Figure 4A). These observations contradict the view that nitrate assimilation through NR action should be more active in the plants to tolerate stress conditions.

The NO is considered a key intracellular signaling molecule, which is involved in diverse developmental and physiological processes (Lamattina et al., 2003; Lamotte et al., 2005), particularly plant responses to abiotic stresses. Nevertheless, the functional roles of NO in salt stress responses remain elusive (Manai et al., 2014). There are two well-characterized enzymatic sources of NO in plants, namely NO synthase (NOS) and NR (Crawford, 2006). The primary function of NR encoded by *NIA1* and *NIA2* is nitrogen assimilation, in which nitrate is converted to nitrite through the activity of NR. Moreover, NR is the key enzyme for NO production in most plants (Tejada-Jimenez et al., 2019). The NR produces NO from nitrite in an NAD(P)H-dependent manner (Chen et al., 2016). This led us to measure the NO content of Col-0 and *nlp7* plants, because at low levels, NO is also known to be a positive regulator of the stress response (Rockel et al., 2002; Del Rio et al., 2004). As shown in Figure 5, the NO content was lower in *nlp7* than in Col-0. In plants, the various physiological functions of NO at the cellular level depend on the specific sites of action, where it is regulated and distributed, and its concentrations in the cell (Hasanuzzaman et al., 2018; Sánchez-Vicente et al., 2019). However, as several other salt stress responses (Figures 8, 9) were suppressed in *nlp7* plants, we could not conclude that NO triggers the salt signaling cascade more effectively in this mutant than in Col-0. In fact, when produced abundantly, the NO can contribute to various ROS-induced cellular responses (Chen et al., 2014). The *nlp7* plants likely produced less ROS, as evidenced by the lower NO accumulation in this mutant than in Col-0. Moreover, the *nlp7* plants appeared to be less stressed than the Col-0 plants under high salinity, as evidenced by the lower levels of MDA (Figure 5A), an indicator of oxidative stress, in the mutant. MDA is a compound with high reactivity in the form of enol. It occurs naturally and is a reliable marker for oxidative stress (Del Rio et al., 2005). Furthermore, the lower proline content (Figure 5B) may be another evidence for the fact that the *nlp7* plants were less stressed than the Col-0 plants. ABA is involved in plant stress response, and its content is increased under stress conditions (Cutler et al., 2010; Fujita et al., 2011; Sah et al., 2016). We observed that *nlp7* plants accumulated less ABA than Col-0 under salt stress (Figure 9A). Moreover, the transcript levels of the ABA biosynthetic gene, *NCED3* were decreased in the *nlp7* plants. Likewise, the transcript levels of *BG1* and *BG2*, which are involved in the conversion of inactive ABA to its active form (Xu et al., 2012; Ng et al., 2014), were reduced in the mutant (Figure 9B). Thus, the lower endogenous ABA levels in *nlp7* plants again likely indicate that this mutant is less stressed than Col-0.

Upon plant exposure to salt stress, high external sodium concentrations lead to the rapid influx of Na^+^ ions into cells through various pathways (Keisham et al., 2018). Typically, the Na^+^ ions enter the cell *via* the K^+^ influx pathway, because the ionic radii of the hydrated form of Na^+^ and K^+^ are similar, making it difficult to discriminate between these two ions (Benito et al., 2014). Consequently, plants growing in highly saline soil often suffer from both Na^+^ toxicity and K^+^ deficiency (Hasegawa et al., 2000). In some halophytes, nitrates are transported to the shoots in a Na^+^-dependent manner (Junfeng et al., 2010; Nie et al., 2015). For instance, in *Beta vulgaris*, Na^+^ improved both nitrate uptake and its transport to the shoots (Kaburagi et al., 2014, 2015). Contrary to our expectation, Na^+^ content was much higher in the *nlp7* plants than in Col-0 plants, particularly in the roots (Supplementary Figure 5). However, both Na^+^ and K^+^ levels were much higher in the *nlp7* plants (Supplementary Figure 5). This finding implies that the *nlp7* plants could accumulate more Na^+^ and K^+^ than Col-0 plants. A possible reason for this phenomenon is that the *nlp7* plants accumulated more nitrates, which are anions, due to their reduced NR activity (Figure 4C), leading to the increased uptake of Na^+^, a cation, from the medium to balance the ionic charges. Under saline conditions, although Na^+^ influx may eventually lead to toxicity for *Arabidopsis* growth, these cations can also serve as an osmoticum (Alvarez-Aragon et al., 2016), although this aspect remains largely undervalued or omissive. Several studies have demonstrated that ionic solute uptake is one of the strategies of plants to adapt to low water potential in salinity environments (Alvarez-Aragon et al., 2016; Genc et al., 2016). For instance, in halophytes, high Na^+^ accumulation contributes to the adaptation to saline environments (Flowers and Colmer, 2015). In these plants, Na^+^ may be used as an osmolyte within the cell. Based on this, the *nlp7* plants may be more tolerant of osmotic stress induced by high salinity. A similar phenomenon has been reported by Alvarez-Aragon and Rodriguez-Navarro (2017). The authors observed that in *Arabidopsis*, nitrates increased the absorption of Na^+^ and its loading into the xylem, resulting in high Na^+^ accumulation in the shoots. Under salt stress, Na^+^ serves an important osmotic function to prevent water loss and plant withering (Alvarez-Aragon and Rodriguez-Navarro, 2017), which supports our conclusion in the present study.

Nitrate assimilation is crucial for vascular plants. Moreover, NR activity is regulated by diverse, intricate mechanisms (for details, refer to the review by Lillo et al., 2004). NR is rapidly inactivated or activated through phosphorylation or dephosphorylation, respectively (MacKintosh and Meek, 2001). In addition, genes encoding NR are regulated at the posttranslational level to modulate enzyme activity in response to various external stimuli (Provan and Lillo, 1999; Lillo et al., 2003). The present study proposes that maintaining the optimal nitrate concentration through the modulation of NR activity may be an effective strategy to improve the salt tolerance of plants. As such, salt tolerance of plants can be enhanced through various mechanisms, and we cannot completely exclude the possibility that *NLP7*, which is rapidly induced under salt stress, acts as a transcriptional regulator of certain unknown genes related to the salt stress tolerance of *Arabidopsis*.

## Data Availability Statement

The original contributions presented in the study are included in the article/Supplementary Material, further inquiries can be directed to the corresponding author.

## Author Contributions

QL conducted nitrate reductase assay, nitrate content assay, ^15^N uptake assay, and proline content measurement and wrote the manuscript. WL conducted plant growth performance tests, chlorophyll assay, protein extraction and bradford assay, and statistical analyses and wrote the manuscript. JC performed MDA content measurements and DTN measured ABA contents. S-AL conducted quantitative RT-PCR. S-WH conducted statistical analyses. HT wrote the manuscript. HL designed the experiments and wrote the manuscript. All authors have reviewed and agreed to the content of the manuscript.

## Conflict of Interest

The authors declare that the research was conducted in the absence of any commercial or financial relationships that could be construed as a potential conflict of interest.

## Publisher’s Note

All claims expressed in this article are solely those of the authors and do not necessarily represent those of their affiliated organizations, or those of the publisher, the editors and the reviewers. Any product that may be evaluated in this article, or claim that may be made by its manufacturer, is not guaranteed or endorsed by the publisher.

## Footnotes

1
http://www.leica-microsystems.com/home/

## Abbreviations

ROS: reactive oxygen species
ABA: abscisic acid
NR: nitrate reductase
NO: nitric oxide
MS: Murashige and Skoog
MDA: malondialdehyde.
