# Supplementary material for: The Loss of Function of the NODULE INCEPTION-Like PROTEIN 7 Enhances Salt Stress Tolerance in Arabidopsis Seedlings
Source: Front Plant Sci. 2022 Jan 24;12:743832. doi: 10.3389/fpls.2021.743832 (PMC8818864; doi:10.3389/fpls.2021.743832)
Supplement: Supplementary file 1 [file Data_Sheet_1.PDF]

Supplementary Material

Supplementary Figures

Figure S1. *nlp7* mutants presented a highly KCl-sensitive phenotype.

(**A**) Four-day old Col-0, *nlp7-1*, and *nlp7-2* seedlings were germinated and grown on media containing 5 mM NO_3^- and then transferred to media containing 5 mM NO_3^- plus 0, 150, or 200 mM KCl. After 2 weeks, each phenotype was confirmed. (**B**) Fresh weight and chlorophyll content of the samples were measured in the phenotype assay. Error bars represent the standard deviation of three independent replicates. Different letters (a, b, or c) within a treatment group indicate significant differences in one-way ANOVA ($P < 0.05$).

Figure S2. Relative transcript levels of *NLP7* in response to KCl treatment.

Relative transcript levels of *NLP7* in the shoots and roots of Col-0 seedlings following treatment with 0, 100, or 200 mM KCl for 6 h. The Col-0 seeds were germinated and grown on nitrogen-free half-strength MS medium containing 5 mM KNO_3 for 9 days and then treated with the indicated media. *AtActin2* was used as the internal control. Error bars represent the standard deviation of three independent replicates. Different letters (a, b, c, or d) within a treatment group indicate significant differences in two-way ANOVA ($P \leq 0.05$, Tukey's test).

Figure S3. Relative transcript levels of *NLP6* in response to salt stress.

Relative transcript levels of *NLP6* in the shoots and roots of Col-0 seedlings following treatment with 0, 100, or 200 mM NaCl for 6 h. The Col-0 seeds were germinated and grown on nitrogen-free half-strength MS medium containing 5 mM KNO_3 for 9 days and then treated with the indicated media. *AtActin2* was used as the internal control. Error bars represent the standard deviation of three independent replicates. Different letters (a or b) within a treatment group indicate significant differences in two-way ANOVA ($P \leq 0.05$, Tukey's test).

Figure S4. Chloride content of *nlp7* under salt stress conditions.

(**A**) Chloride concentration in the shoots and roots of 9-day-old Col-0, *nlp7-1*, and *nlp7-2* seedlings. The seeds were germinated and grown on nitrogen-free half-strength MS medium containing 5 mM KNO_3 and then treated with 0, 150, or 200 mM NaCl for 24 h. Error bars represent the standard deviation of three independent replicates. Different letters (a, b, c, d, e, or f) within a treatment group indicate significant differences in two-way ANOVA ($P \leq 0.05$, Tukey's test).

Figure S5. Na^+ and K^+ content following NaCl treatment.

The Na^+ and K^+ content in the shoots and roots of 9-day-old Col-0, *nlp7-1*, and *nlp7-2* seedlings. The seeds were germinated and grown on nitrogen-free half-strength MS medium containing 5 mM KNO_3 and then treated with 200 mM NaCl for 6–24 h. The Na^+ translocation from the roots to the shoots was calculated by the measurement of the Na^+ and K^+ content of the roots and shoots using ICP-OES (Agilent) based on a standard curve obtained with Na^+ and K^+ standard solutions. Error bars represent the standard deviation of three independent replicates. Different letters (a, b, or c) within a treatment group indicate significant differences in two-way ANOVA ($P \leq 0.05$, using Tukey's test).

Figure S6. Relative transcript levels of *HY5* under salt stress.

Relative transcript levels of *HY5* in the shoots and roots of Col-0 seedlings following treatment with 0, 100, or 200 mM NaCl for 6 h. The Col-0 seeds were germinated and grown on nitrogen-free half-strength MS medium containing 5 mM KNO_3 for 9 days and then treated with the indicated media. *AtActin2* was used as the internal control. Error bars represent the standard deviation of three independent replicates. Different letters (a or b) within a treatment group indicate significant differences in two-way ANOVA ($P \leq 0.05$, Tukey's test).

Figure S7. Phenotype of the wildtype Col-0 plants under fluridone-induced salt stress.

(**A**) Comparison of the growth performance of Col-0 and *nlp7* under salt stress induced by different concentrations of fluridone. Four-day-old Col-0, *nlp7-1*, and *nlp7-2* seedlings were germinated and grown on nitrogen-free half-strength MS medium containing 5 mM KNO_3 and then transferred to media supplemented with 200 mM NaCl combined with various concentrations of fluridone (0, 0.05, 0.5, 1, 5, and 20 µM). After 2 weeks, each phenotype was confirmed. (**B**) Quantification of the primary root length and fresh weight of the test seedlings in response to salt stress. Error bars represent the standard deviation of three biological replicates. Different letters (a, b, c, or d) within a treatment group indicate significant differences in two-way ANOVA ($P \leq 0.05$, Tukey's test).

Supplementary Table S1: List of primers used in this study

Gene name	Primer sequence (5' – 3')	Note
NLP7-Full	F: ATGTGCGAGCCCGATGATAATTC<br>R: TCACAATTCTCCAGTGCTCTCGCAG	*NLP7* full lengh primers
NLP7-proGUS	F: GGGCCAACTATAGAGGAATGGT<br>R: ACAATACAACTGTGCCCCAAAT	*NLP7* promoter fragment primers
LBb1.3	F: ATTTTGCCGATTTCGGAAC	Left border gene forward primer
NLP7	F: GCTGAAAGTTGATGCAGGAACG<br>R: CAGGAGCTCCCTAGATTTGTCG	qPCR primers
NLP6	F: ATGGAACTTGACGACTTGGATCTCA<br>R: TCACAAGCACATCATAGTTTCCTCTGA	qPCR primers
NRT1.1	F: TAAGGGATCAGGAAGCGGGA<br>R: AAGAGGATGCATGTTGCCCA	qPCR primers
HY5	F: CAC TAC AGC CGG TAT GCA AG<br>R: CGA TCC TAA ACC AAA CCC TTC	qPCR primers
COR47	F: ACACCAACGGTCGCAACA<br>R: TCCACGATCCGTAACCTCTGT	qPCR primers
RD29A	F: GATATCGACAAGGATGTGCCG<br>R: GTATCCAGGTCTTCCCTTCGC	qPCR primers
NRT1.5	F: CGGACTTGTGATTGCTGTCATAGC<br>R: GGAGCCTGCCAGAAGATGCTT	qPCR primers
NRT1.8	F: GGCTTCAGATTCTTGGATAGAGC<br>R: AACCACAGAGTAGAGGATGGTGC	qPCR primers
NCED3	F: GCTGCGGTTTCTGGGAGAT<br>R: TTGAGAAGACGATAATGGCGG	qPCR primers
BG1	F: TTACTATACTTCAGTGTTTGCAAAAG<br>R: CTAGAGTTCTTCCCTCAGCTTG	qPCR primers

BG2	F: AATGGGATGGACGACATCGA<br>R: CCGTAATCGTTAGTAGCTTCCGTTA	qPCR primers
