# Supplementary figures and images for: The Loss of Function of the NODULE INCEPTION-Like PROTEIN 7 Enhances Salt Stress Tolerance in Arabidopsis Seedlings
Source: Front Plant Sci. 2022 Jan 24;12:743832. doi: 10.3389/fpls.2021.743832 (PMC8818864; doi:10.3389/fpls.2021.743832)

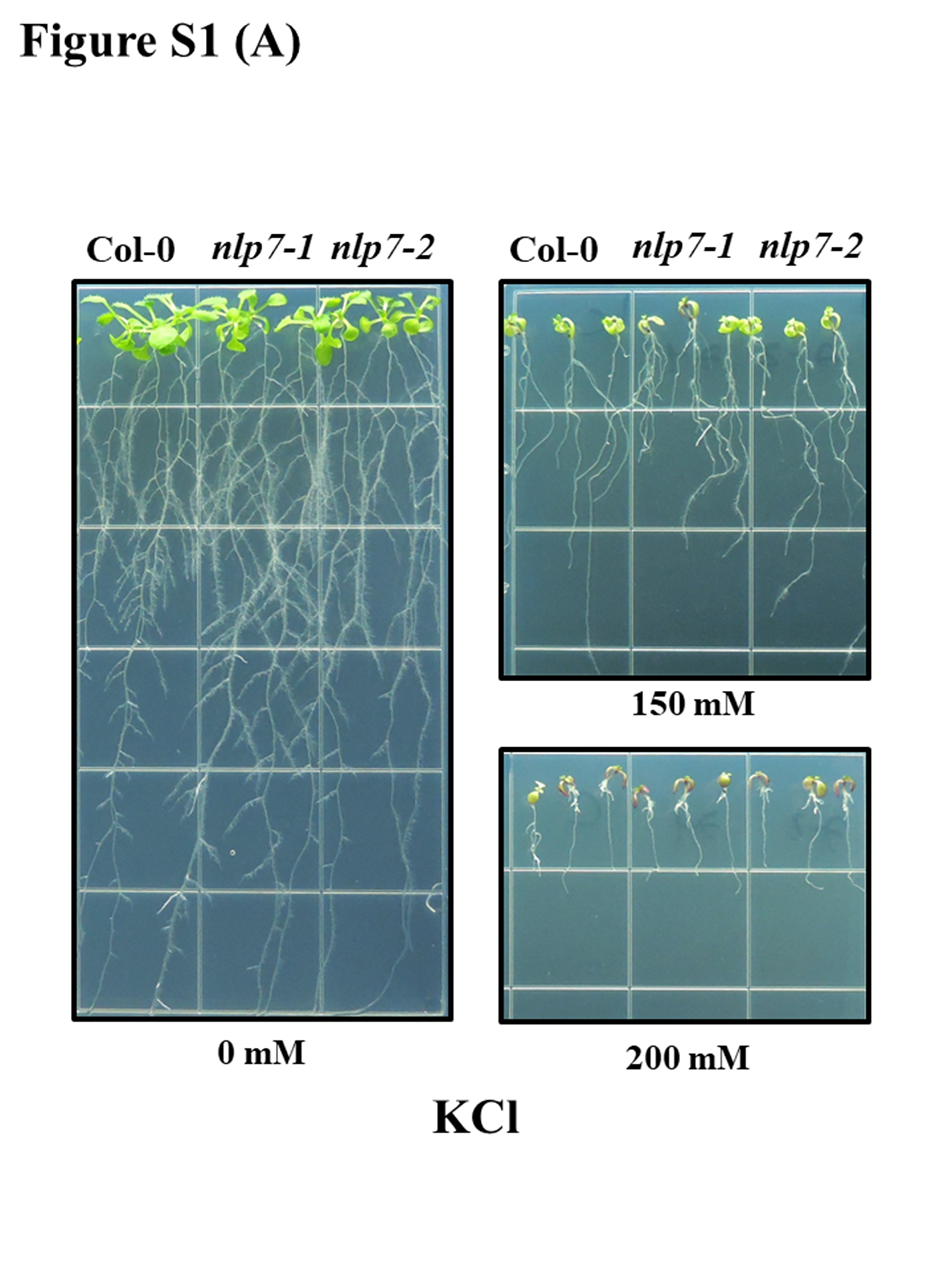

Supplement: Supplementary file 2 [file Image_1.TIF]

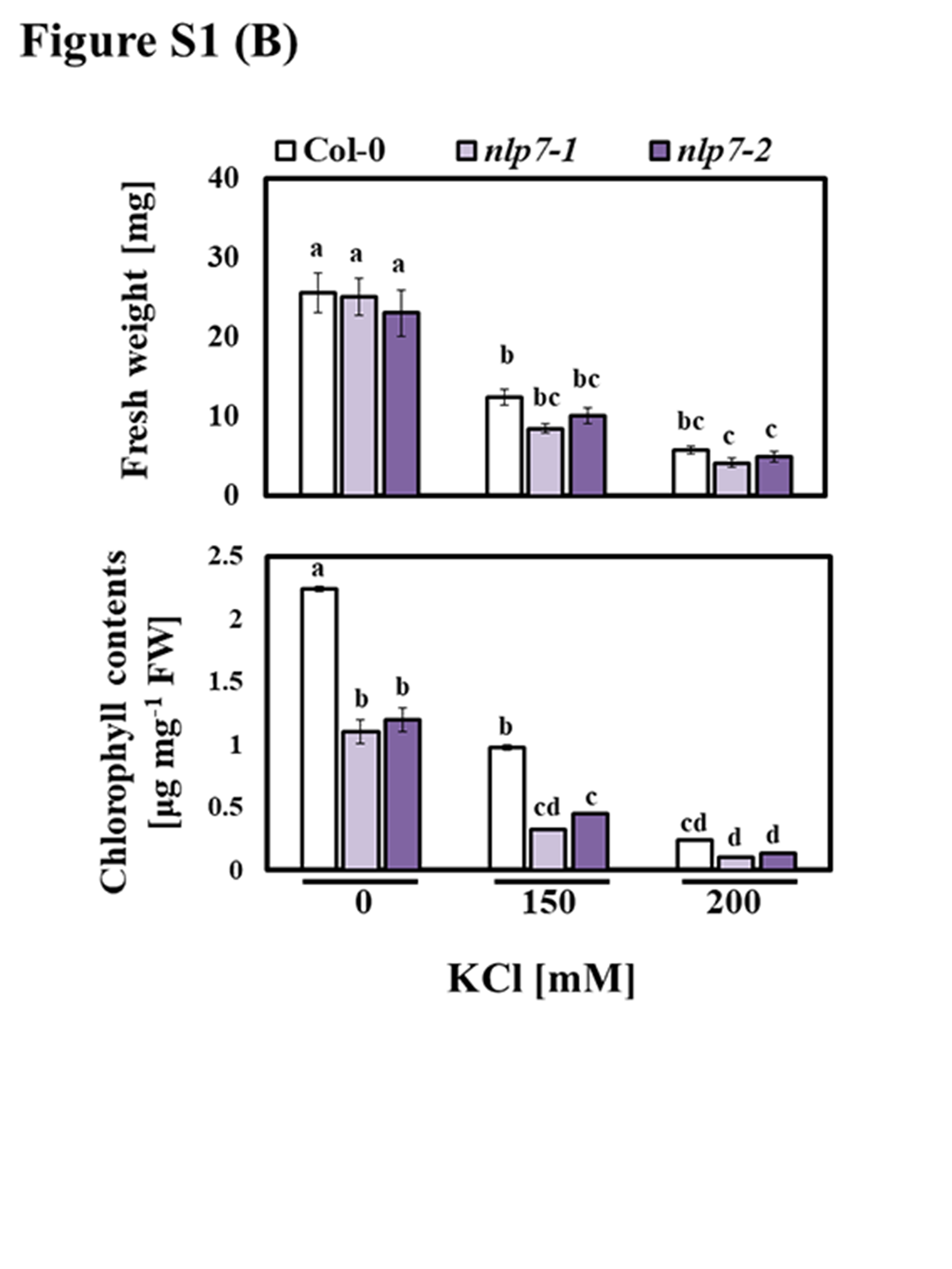

Supplement: Supplementary file 3 [file Image_2.TIF]

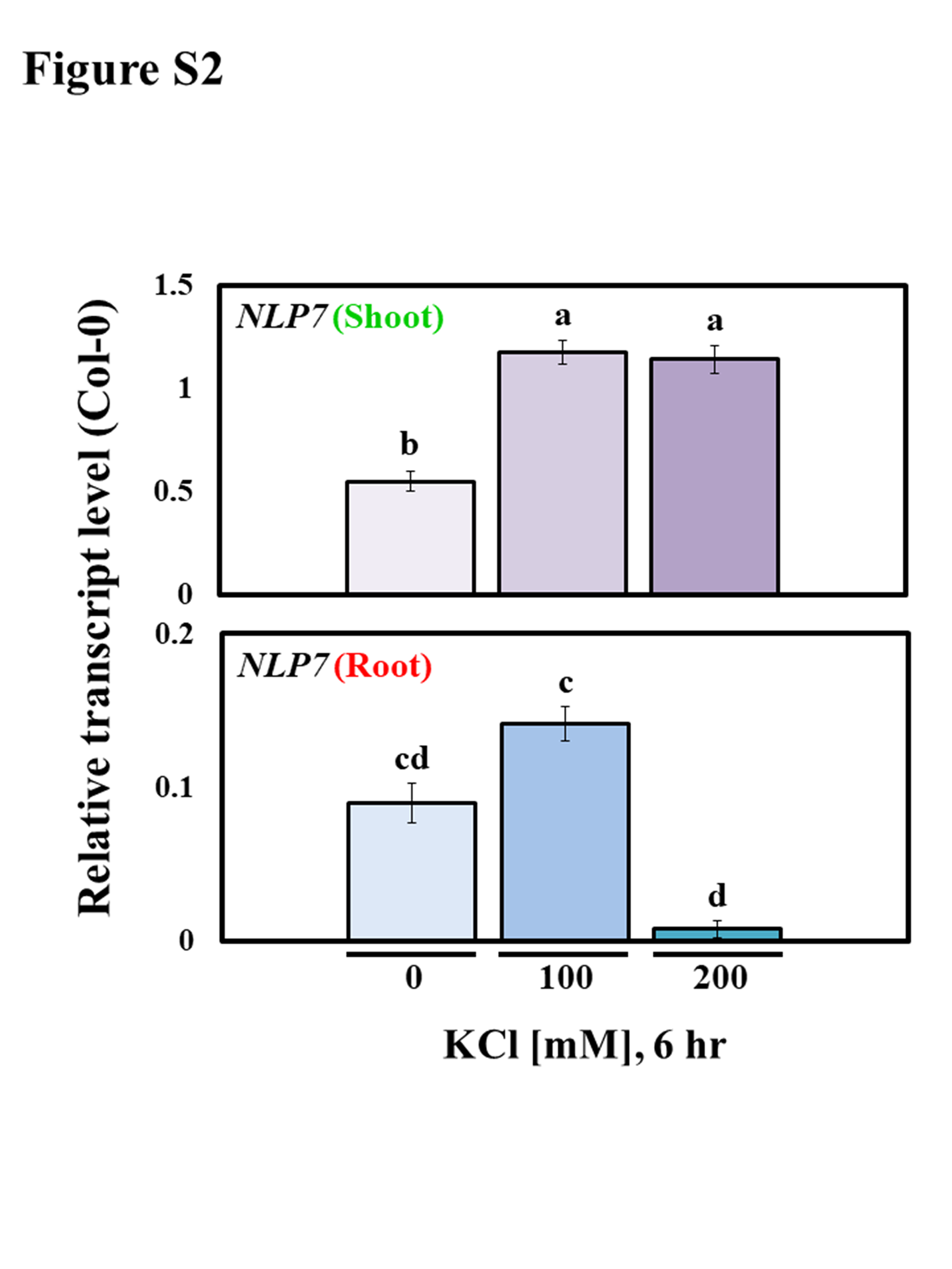

Supplement: Supplementary file 4 [file Image_3.TIF]

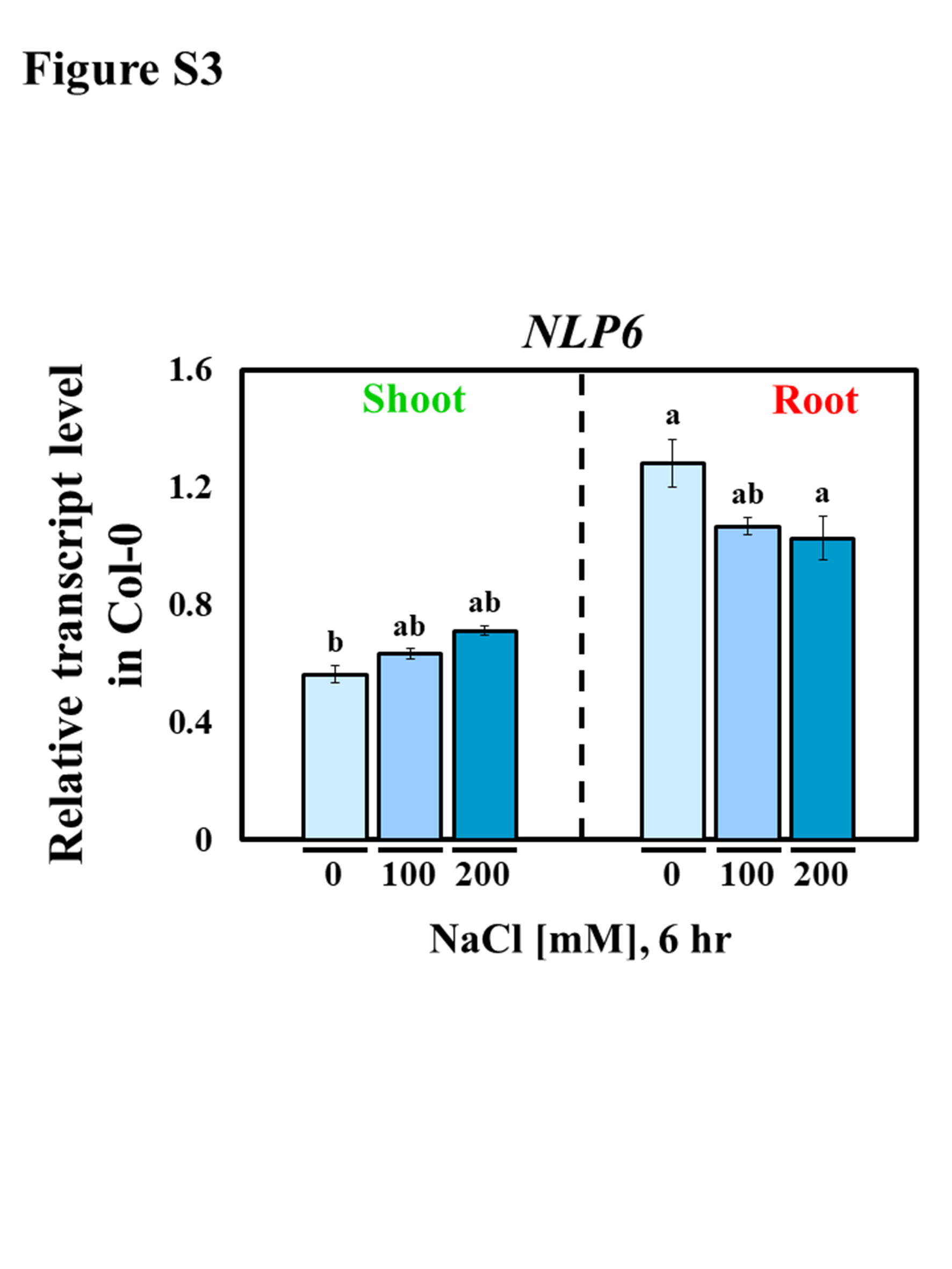

Supplement: Supplementary file 5 [file Image_4.TIF]

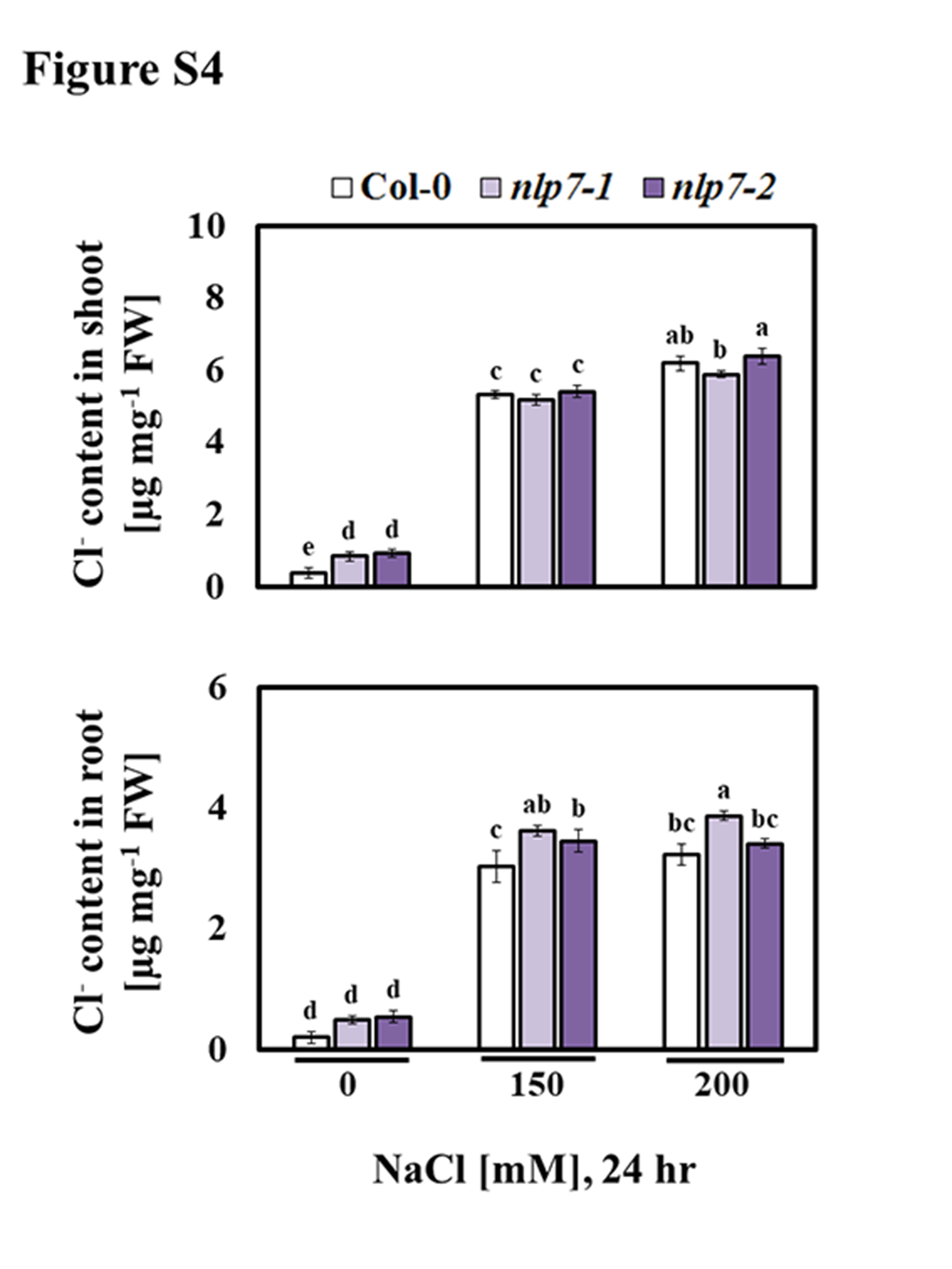

Supplement: Supplementary file 6 [file Image_5.TIF]

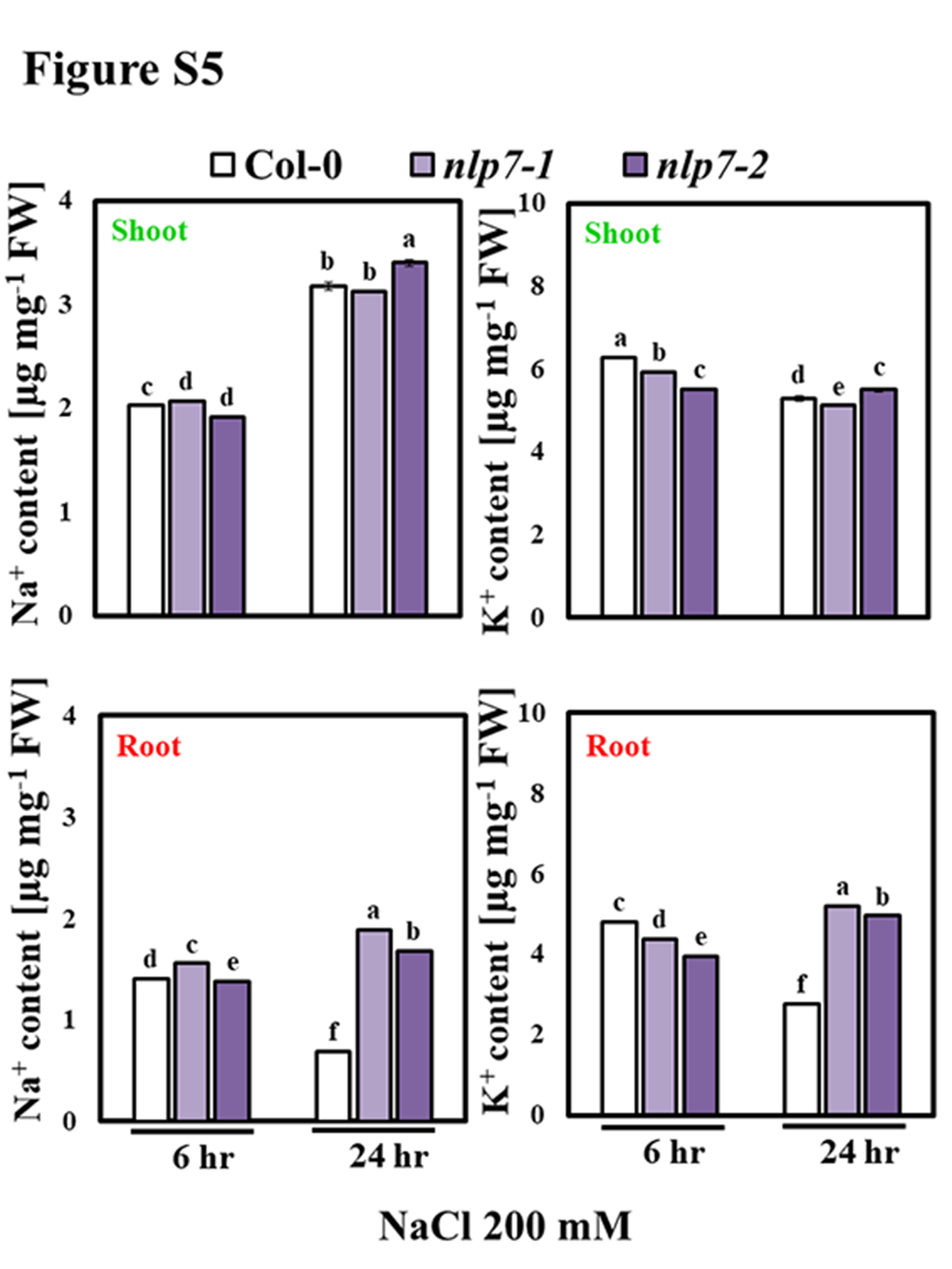

Supplement: Supplementary file 7 [file Image_6.TIF]

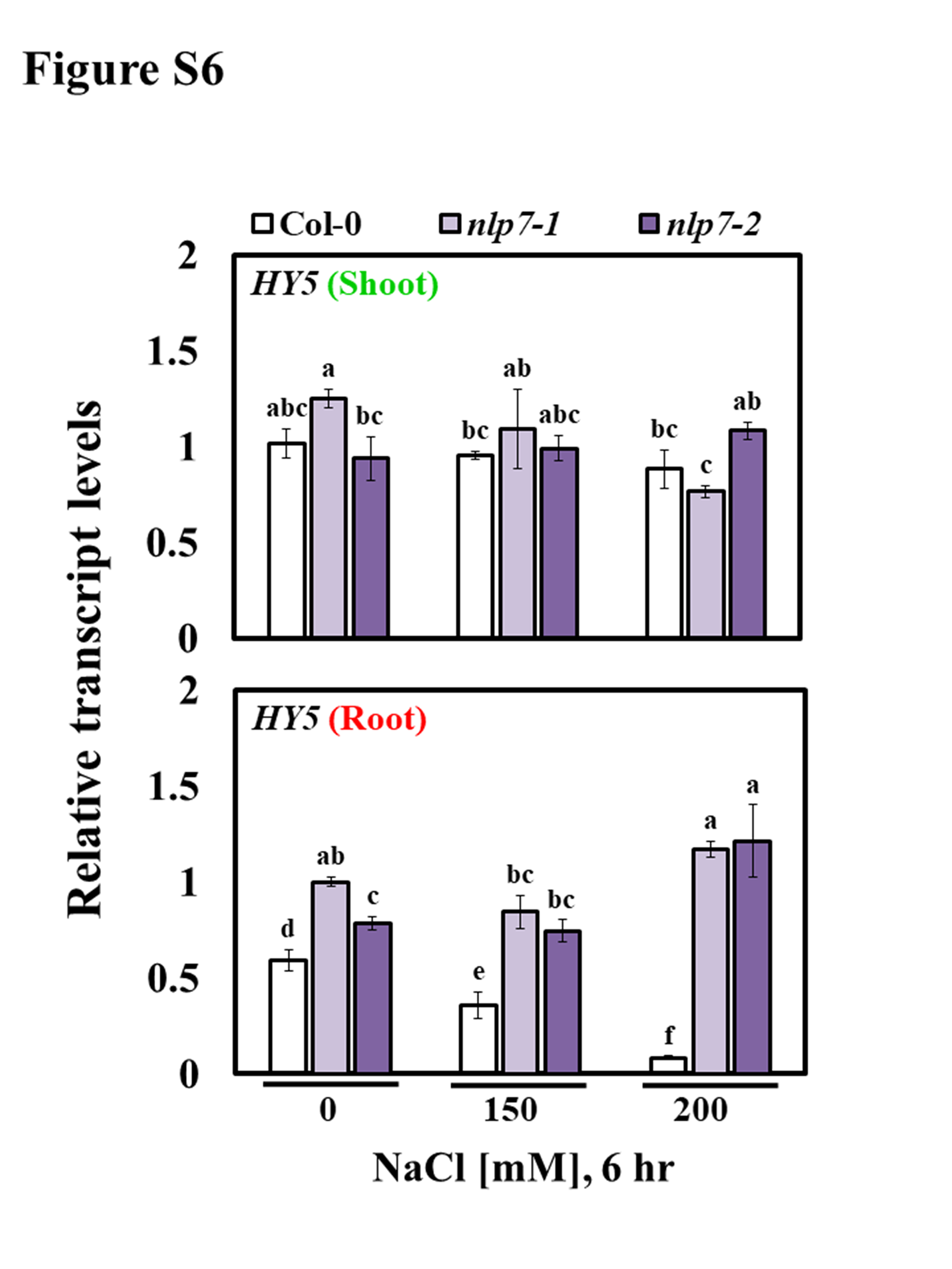

Supplement: Supplementary file 8 [file Image_7.TIF]

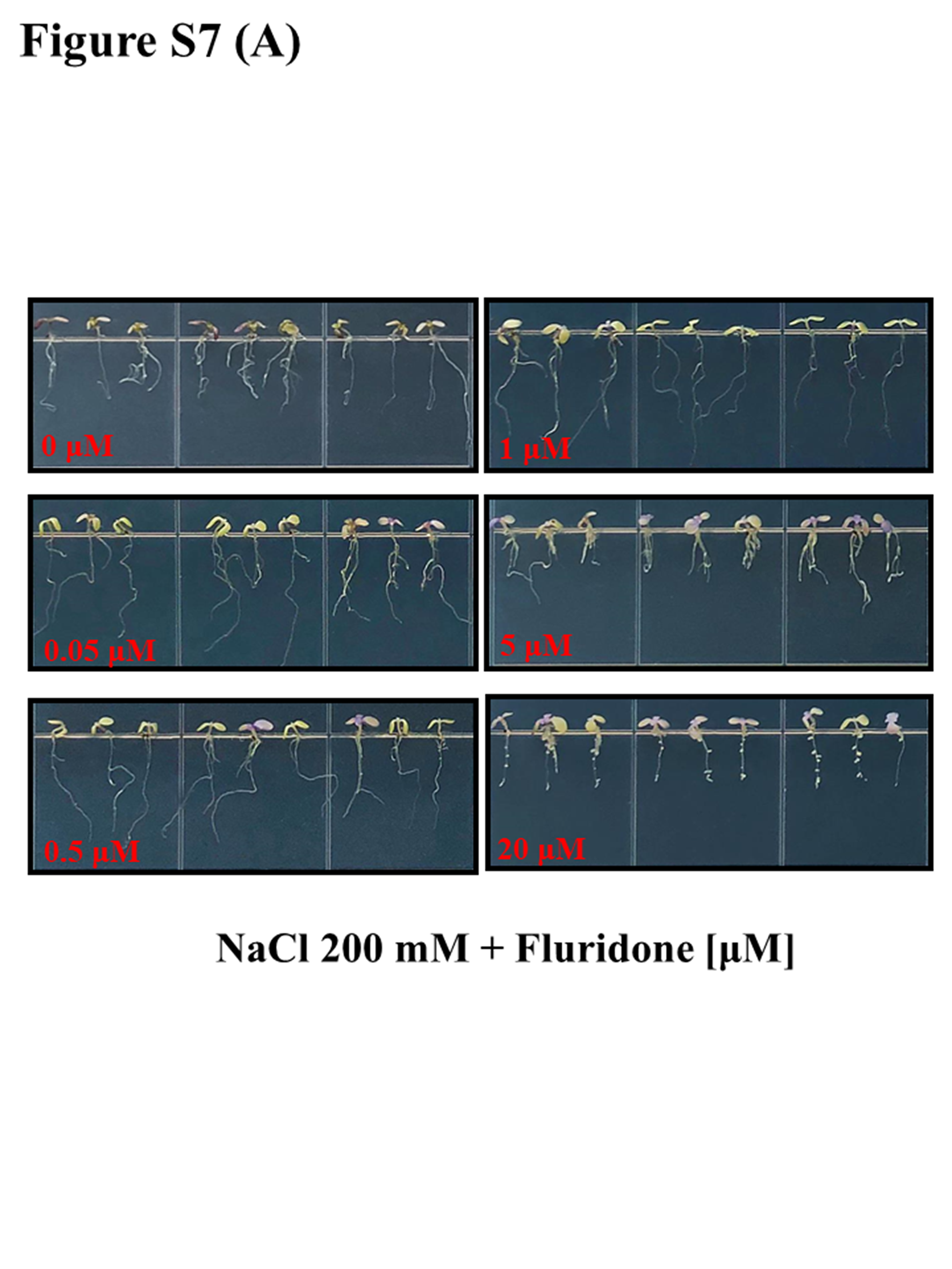

Supplement: Supplementary file 9 [file Image_8.TIF]

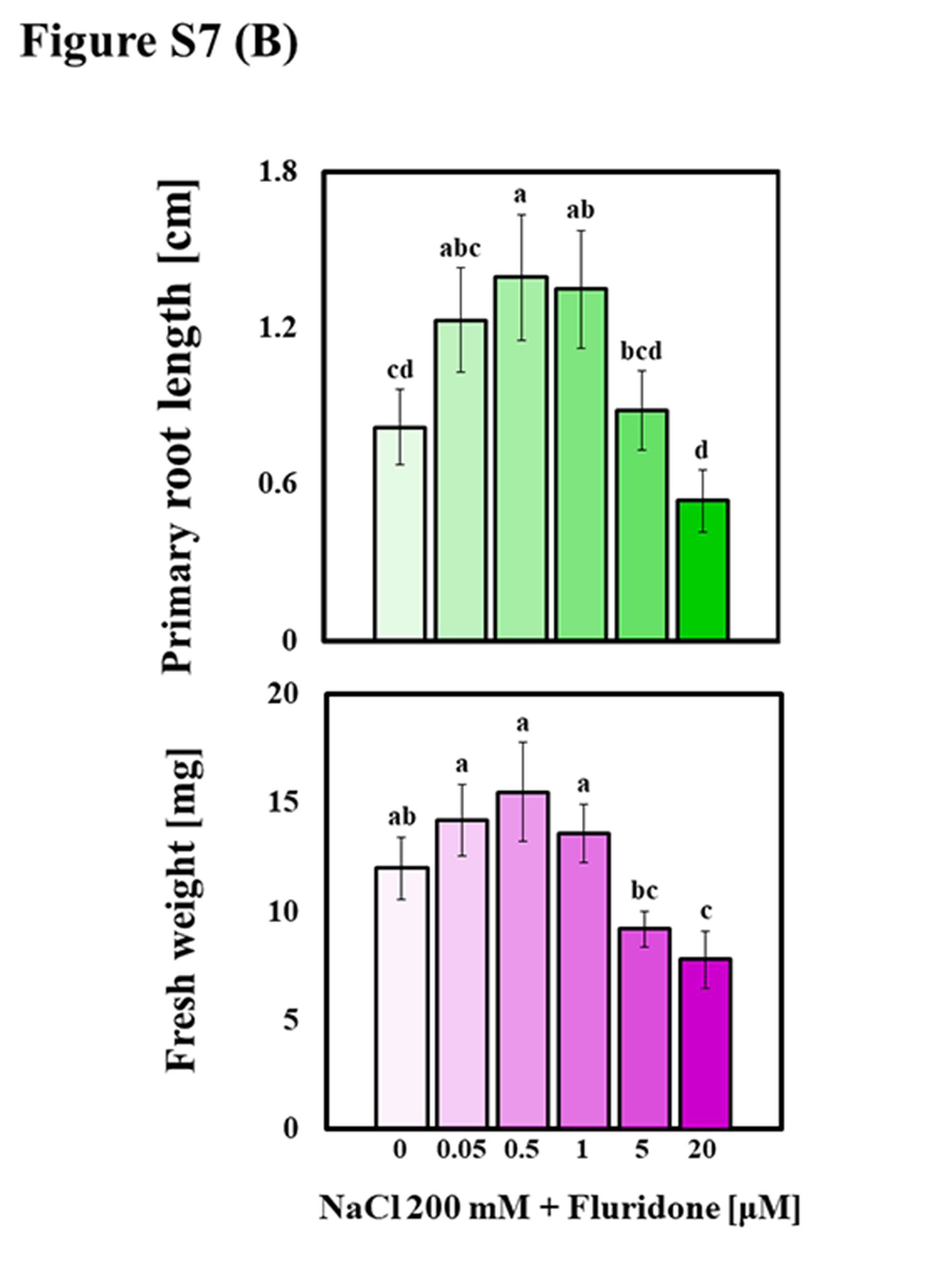

Supplement: Supplementary file 10 [file Image_9.TIF]
